# The FUSED LEAVES1‐*ADHERENT1* regulatory module is required for maize cuticle development and organ separation

**DOI:** 10.1111/nph.16837

**Published:** 2020-08-27

**Authors:** Xue Liu, Richard Bourgault, Mary Galli, Josh Strable, Zongliang Chen, Fan Feng, Jiaqiang Dong, Isabel Molina, Andrea Gallavotti

**Affiliations:** ^1^ Waksman Institute of Microbiology Rutgers University Piscataway NJ 08854‐8020 USA; ^2^ Department of Biology Algoma University Sault Ste. Marie ON P6A 2G4 Canada; ^3^ School of Integrative Plant Science Plant Biology Section Cornell University Ithaca NY 14853 USA; ^4^ Department of Plant Biology Rutgers University New Brunswick NJ 08901 USA

**Keywords:** ADHERENT1 (AD1), cuticle, FUSED LEAVES1 (FDL1), maize, MYB, transcriptional regulation

## Abstract

- All aerial epidermal cells in land plants are covered by the cuticle, an extracellular hydrophobic layer that provides protection against abiotic and biotic stresses and prevents organ fusion during development.
- Genetic and morphological analysis of the classic maize *adherent1* (*ad1*) mutant was combined with genome‐wide binding analysis of the maize MYB transcription factor FUSED LEAVES1 (FDL1), coupled with transcriptional profiling of *fdl1* mutants.
- We show that *AD1* encodes an epidermally‐expressed 3‐KETOACYL‐CoA SYNTHASE (KCS) belonging to a functionally uncharacterized clade of KCS enzymes involved in cuticular wax biosynthesis. Wax analysis in *ad1* mutants indicates that *AD1* functions in the formation of very‐long‐chain wax components. We demonstrate that FDL1 directly binds to CCAACC core motifs present in *AD1* regulatory regions to activate its expression. Over 2000 additional target genes of FDL1, including many involved in cuticle formation, drought response and cell wall organization, were also identified.
- Our results identify a regulatory module of cuticle biosynthesis in maize that is conserved across monocots and eudicots, and highlight previously undescribed factors in lipid metabolism, transport and signaling that coordinate organ development and cuticle formation.

## Introduction

The plant cuticle provides protection against environmental stresses and represents one of the most important adaptations for terrestrial plants (Yeats & Rose, 2013). In addition, the cuticle plays an essential role in development, ensuring that growing organs in tightly appressed structures (i.e. flowers, embryos) do not fuse together (Javelle *et al*., 2011; Ingram & Nawrath, 2017). The cuticle is synthesized by the epidermis and consists of several layers. The innermost layer, which directly contacts the epidermal cell wall, is composed of a cutin matrix, containing a mix of polyesters comprising mainly glycerol and long‐chain (C16 and C18) hydroxy fatty acids (Kolattukudy, 1980; Graca *et al*., 2002). Embedded within this layer are intracuticular waxes. Waxes also form an outermost layer, the epicuticular wax layer, that covers the cutin matrix, either as a thin film or as diversely shaped wax crystals that can be seen on organ surfaces. Collectively, intracuticular and epicuticular waxes are termed cuticular waxes (Buschhaus & Jetter, 2011; Lee & Suh, 2015a). Cuticular waxes are composed of complex mixtures of very long chain fatty acids (VLCFAs; C24 to C34) and their derivatives (Javelle *et al*., 2011). The biosynthesis of VLCFA is carried out by the FATTY ACID ELONGASE (FAE) complex localized in the endoplasmic reticulum (ER). The FAE complex consists of four enzymes, including 3‐KETOACYL‐CoA SYNTHASE (KCS), which catalyze the first rate‐limiting step in the elongation process and confers substrate chain length specificity (Joubes *et al*., 2008). Subsequently, VLCFAs are transformed into primary alcohols and wax esters, or into aldehydes, alkanes, secondary alcohols and ketones (Samuels *et al*., 2008), and are exported to the apoplast by transporters at the plasma membrane (Yeats & Rose, 2013).

The biosynthetic pathways and gene networks underlying cuticle formation have been identified based predominantly on mutant analysis in Arabidopsis and characterization of maize *glossy* mutants. In addition to the cuticle defects observed in these mutants, many also show organ fusion defects (Ingram & Nawrath, 2017). Studies in Arabidopsis have also shown that several cuticular wax biosynthesis genes are transcriptionally regulated by members of the AP2/ERF and MYB transcription factor (TF) families. The latter includes AtMYB30, AtMYB94 and AtMYB96 (Raffaele *et al*., 2008; Seo *et al*., 2011; Lee & Suh, 2015b). Characterization of cuticle defects in the maize *fused leaves1/*Zm*myb94* mutant (La Rocca *et al*., 2015), encoding a close homolog of AtMYB94, indicates conservation of the MYB94 regulatory pathway, although the lack of organ fusion defects in At*myb94* mutants suggests species‐specific aspects of regulation.

Cuticle structure, composition, and permeability vary widely among plant species and tissue types (Petit *et al*., 2017), likely reflecting the vastly different environmental challenges that plants experience in natural conditions. As the first line of defense against external factors, cuticle characteristics can confer beneficial traits such as disease resistance and drought tolerance, and they are considered a potential target for breeding improved varieties (Lin *et al*., 2020). Therefore, understanding gene networks involved in cuticle formation in diverse species is critical, in particular in crop species such as maize. This also includes knowledge about how cuticle formation is coordinated with cell division and expansion such that ectopic organ fusion is prevented. Here we characterize the interaction between *AD1* and *FDL1*, two key genes for maize cuticle development, and identify many potential direct targets of FDL1 function. Ultimately, this information could be used to generate stress tolerant crop varieties without impacting plant growth.

## Materials and Methods

### Plant materials and phenotyping

The *ad1‐224* allele, originally identified in an ethyl methanesulfonate (EMS) mutagenesis screen, was back‐crossed to A619 before phenotypic measurements. The *ad1‐ref* allele (*ad1‐109D* and *ad1‐110E*) and *FDL1* insertion line mu1092890 (UFMu‐13110) were obtained from the Maize Genetics Cooperation Stock Center, while the *ad1‐9.2121* allele was discovered in an EMS screen of a *ramosa1* mutant. The phenotype of single and double mutant plants was analyzed in field‐ and glasshouse‐grown plants. Student's *t*‐test was used to determine statistical significance for all measurements.

### Positional cloning

An F_2_ population was generated by crossing the *ad1‐224* mutant from the original M2 genetic background to the B73 inbred line. Using PCR‐based molecular markers, we mapped *ad1* to a 10.8 Mb window on chromosome 1 between markers umc1147 and umc2080. For bulked segregant whole genome sequence analysis, a single bulk genomic DNA sample was obtained from 10 *ad1* plants. Library preparation and sequencing of the sample was performed by Psomagen Inc. (Rockville, MD, USA). 150 bp pair end sequencing on a NovaSeq 6000 (Illumina, San Diego, CA, USA) produced fastq files that were analyzed using a published pipeline (Dong *et al*., 2019) and B73v3 as the reference genome. Single nucleotide polymorphisms (SNPs) within the 10.8 Mb *ad1* mapping window were analyzed in IGV (Robinson *et al*., 2011).

### Microscopy

For light microscopy, 8 µm tissue sections were stained with Safranin O/Alcian Blue, and images were acquired using a Leica DM5500B microscope equipped with a Leica DFC450 C digital camera (Leica Microsystems Inc., Buffalo Grove, IL, USA). For organ fusion defects and epicuticular wax crystals, fresh tissue was imaged using a Jeol JCM‐6000PLUS Scanning Electron Microscope (SEM).

For confocal microscopy, we cloned the full‐length coding sequences of *AD1* into the pEarly‐Gate 104 vector (YFP‐AD1), and of Arabidopsis *CNX1* into the pEarly‐Gate mCHERRY vector (mCHERRY‐CNX1). YFP‐AD1 and mCHERRY‐CNX1 were transiently expressed in *Nicotiana* *benthamiana* leaves using Agrobacterium‐mediated injections. Images were obtained on a Leica SP5 confocal microscope. The YFP and mCHERRY signals were acquired using 514, 520–575 and 594, 625–655 nm excitation and emission settings, respectively, and images were analyzed using fiji (https://fiji.sc).

### Expression analysis

Total RNA from embryos and endosperm at 10 d after pollination, juvenile (3^rd^ seedling leaf) and mature leaves (9^th^ leaf), seedling roots, and tassel and ear at 0.2 and 0.6 cm stages was extracted using the RNeasy Plant Mini Kit (Qiagen). Retrotranscription was performed using the qScript cDNA Synthesis kit (Quantabio, Beverly, MA, USA). cDNA was amplified with PerfeCTa^®^ SYBR^®^ Green FastMix^®^ (Quanta Biosciences, Quantabio) on an Illumina Eco Real‐Time PCR System. Relative expression levels were calculated using the 2^−ΔΔCt^ method with *UBIQUITIN* as control. Primers are listed in Supporting Information Table S1.

For *in situ* hybridizations, samples were dissected and fixed using paraformaldehyde acetic acid. Hybridizations were performed at 59°C. Samples were treated with anti‐digoxygenin (DIG) antibody (Roche) and signals were detected using NBT/BCIP (Promega). The 3’UTR of *AD1* and *FDL1* were PCR amplified (primers provided in Table S1), cloned into pENTR223.1‐Sfi, and used to synthesize antisense probes using T7 RNA polymerase (Promega), after digestion with EcoRI. Sense probes were PCR amplified from the same clones using primers provided in Table S1.

### Phylogenetic analysis

Full‐length amino acid sequences of the KCS family were obtained from Phytozome 12 and aligned using clustal omega (https://www.ebi.ac.uk/Tools/msa/clustalo/). The alignment file was then used to generate a neighbor‐joining rooted tree with Mega 5.0, applying the Bootstrap method and 1000 bootstrap replications (Simmons & Freudenstein, 2011).

### Water loss and Chl leaching assays

For water loss assays, leaves of 3‐wk old dark‐acclimated plants were excised and soaked in water for 60 min in the dark. Excess water was removed from the leaves, and 3 cm pieces of leaves were weighed at different time points using a precision balance. Three measurements were averaged per time point.

Chlorophyll leaching assays were performed on leaves of 3‐wk‐old plants. 2 g of each sample were incubated on ice for 30 min and immersed in 30 ml of 80% ethanol at room temperature. Aliquots of 100 µl were removed from the solution every 15 min after immersion. Extracted Chl was quantified by measuring absorbance at 647 and 664 nm using a spectrophotometer. Three measurements were averaged for each time point.

For the drought stress experiment in Fig. S6, seedlings were grown in pots and watered up to the fourth leaf stage, then plants were not watered for 30 d. The experiment was repeated twice (*n* = 18).

### Toluidine blue permeability test

Toluidine blue tests were performed as previously described (Tanaka *et al*., 2004; Doll *et al*., 2020). Two‐day‐old etiolated coleoptiles were stained for 5 min in a toluidine blue solution (0.05% w/v) with Tween 20 (Thermo Fisher Scientific, Waltham, MA, USA) (0.1% v/v) and washed in tap water. For quantification, five coleoptiles were excised, placed in tubes containing 1 ml of 80% ethanol, and incubated for 4 h in the dark until all dye and Chl had been extracted. Absorbance of the solution was detected using a spectrophotometer. Five repeats were performed per treatment.

### Cuticular wax analysis

Waxes were extracted from an expanded portion of juvenile leaves of *ad1* (3^rd^ leaf) mutants. A small section of each leaf was preliminary scanned by SEM to verify loss of crystal waxes in the mutants. Wax components were identified by GC‐MS and quantified by gas chromatograph coupled to a flame ionization detector (GC–FID) as previously described (Bourgault *et al*., 2020).

### DAP‐seq

DAP‐seq samples were performed using the TNT rabbit reticulocyte system (Promega) as previously described using 1 μg of genomic B73 DNA library (Bartlett *et al*., 2017; Galli *et al*., 2018). Sequenced reads were mapped to the B73v3 genome. Peaks were called using Gem v.2.5 using a GST‐HALO negative control sample for background subtraction and an FDR of 0.00001 (‐‐q 5). Peaks were associated with their closest putative target genes using chipseeker (Yu *et al*., 2015).

### Electrophoretic mobility shift assays (EMSAs)

For EMSAs, region −411 to −233 relative to the *AD1* start codon (+1) within the *AD1* promoter was biotinylated using the Biotin 3′ End DNA Labeling Kit (Thermo Fisher Scientific). DNA binding assays were performed using the Lightshift Chemiluminescent EMSA kit (Thermo Fisher Scientific): binding reactions containing 1X Binding Buffer, 50 ng μl^–1^ polydI/dC, 2.5% glycerol, 2 μl biotinylated probe, and 1 μl purified GST‐FDL1 protein were incubated at room temperature for 20 min and loaded on a 6% DNA retardation gel (Thermo Fisher Scientific) before transfer to a nylon membrane. Detection was carried out according to the manufacturer's recommendations. Competition with unlabeled probe was carried out using 50‐fold excess of unlabeled probe. A mutated probe was generated by annealing two complementary oligos in which two core CCAACC elements (1 and 2, Fig. S7) were changed to TTTTTT, and one element was changed to TTTTTTTTTTTT (3, Fig. S7). Primers are listed in Table S1.

### Transient transcription dual‐LUC assay

The isolation of B73 maize mesophyll protoplasts, PEG‐calcium transfection of plasmid DNA, and protoplast culture were performed as described previously (Sheen, 2001). The effector vector pRI101 was used for the expression of the *FDL1* gene driven by a 35S promoter. The *AD1* promoter was cloned in the reporter vector pGreenII 0800‐LUC. The same mutations in the CCAACC elements used in EMSA were introduced in the *AD1* promoter. The ratio of LUC : REN activity was measured using the dual luciferase reporter (DLR) assay system (Promega). Primers are listed in Table S1.

### RNA‐seq analysis

Coleoptiles of seedlings grown in a 28°C growth chamber were collected 3 d after germination from homozygous and heterozygous *fdl1‐Mu* plants in the original W22 background. Three biological replicates of five 0.3–0.4 cm coleoptile tips from wild‐type and *fdl1* mutants were collected, and RNA was extracted using the RNeasy Plant Mini Kit (Qiagen). Libraries were processed using a TruSeq stranded mRNA kit and sequenced on an Illumina NovaSeq 6000 platform (Psomagen Inc.). Sequenced reads were mapped to the B73v3 genome using tophat, and differential gene expression was determined using cuffdiff (Trapnell *et al*., 2012).

## Results

### The *adherent1* mutant shows abnormal fusions between organs and cells

In search of modifiers of the *ramosa1 enhancer locus2* (*rel2*) mutant, we performed an EMS enhancer/suppressor screen of *rel2‐ref* mutants in the A619 genetic background (Liu *et al*., 2019). Within family M2‐92‐224 we observed a severe upright tassel branch phenotype segregating as a single recessive locus (Fig. S1). Subsequent genetic analysis showed that this mutant phenotype was independent of the *rel2‐ref* mutation and strongly resembled a classic maize mutant, isolated a century ago, called *adherent1* (*ad1*) (Kempton, 1920). Complementation tests between the M2‐92‐224 mutant and previously isolated *ad1* alleles (*ad1‐109D* and *ad1‐110E*) indicated that M2‐92‐224 was a new allele of *ad1*, which we renamed *ad1‐224*.

**Fig. 1:**
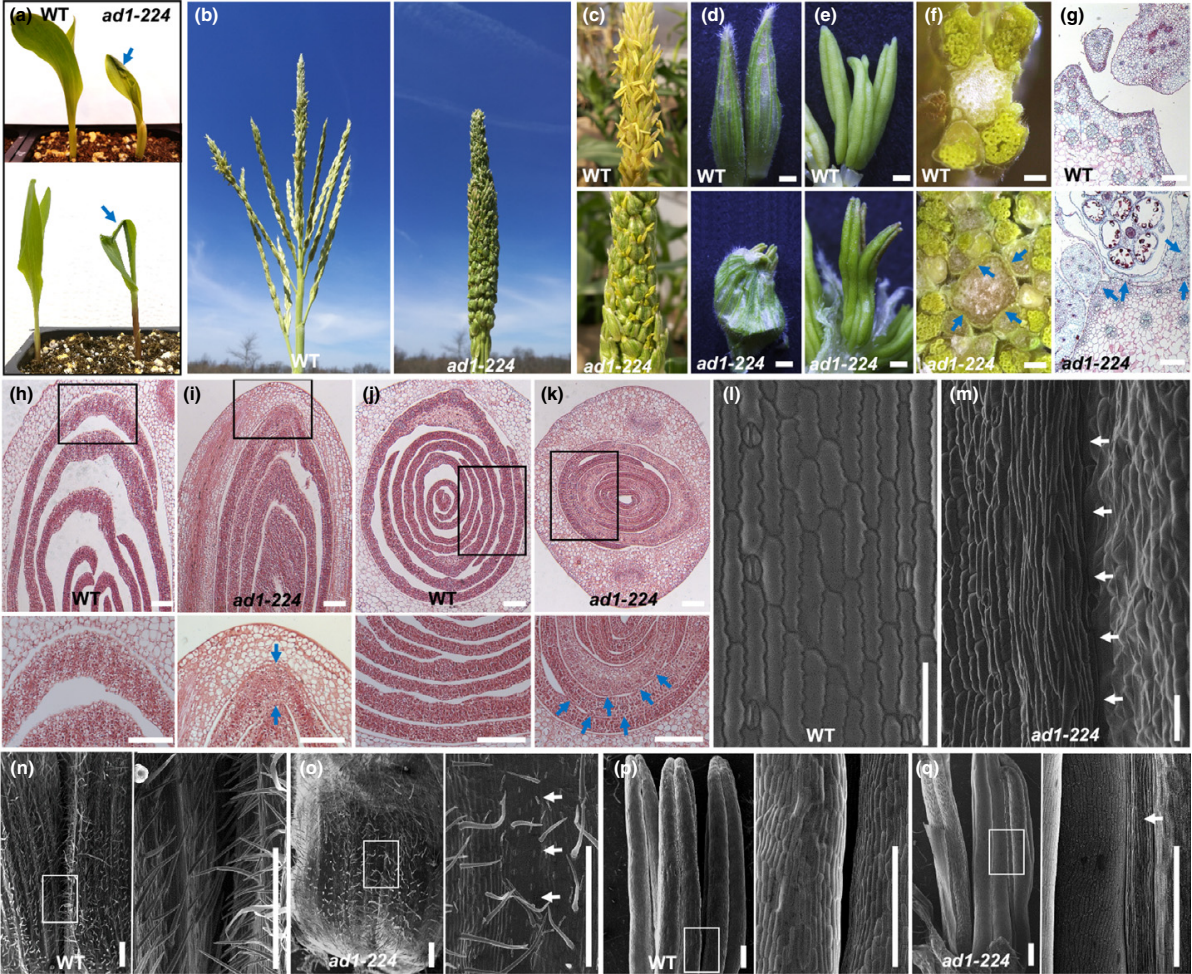
The maize *adherent1* mutant shows organ fusion defects. (a) The seedling phenotype of wild‐type and *ad1‐224* plants. The second leaf is rolled and fused to the adaxial surface of the first leaf (upper image). The second leaf and first leaf show fusion at the leaf tip (lower image). Blue arrows point to fused leaves. (b) Mature tassel phenotype. Tassel branches are fused together in *ad1‐224*. (c, d) The spikelets of *ad1* tassels adhere to each other, and the glumes of adjacent spikelets are fused together. (e) Anthers in the same floret show fusion in *ad1‐224*. (f) Cross sections of wild‐type and *ad1‐224* mature tassels. (g) Saffranin‐O Alcian Blue staining of transverse sections of wild‐type and *ad1‐224* mature tassel. (h–k) Longitudinal and transverse sections of wild‐type (h, j) and *ad1‐224* (i, k) germinating seedlings. Higher magnification (lower image) of the area framed in upper image. Blue arrows, fused regions. (l, m) SEMs of wild‐type leaves (l) show no fusion defects, while a seedling leaf blade is curled up and fused to itself in *ad1‐224* mutants (m). (n, o) Spikelet phenotype of wild‐type and *ad1‐224* mutants. Note that the glumes of adjacent spikelets are separated in wild‐type but fused in *ad1‐224* mutants. Higher magnification (right image) of the area framed in left image. (p, q) Anthers of the same floret are separated in wild‐type tassels, but fused in *ad1‐224* mutants. Higher magnification (right image) of the area framed in the left image. Arrows (in m, o, q) point to regions of fusion. Bars: (c–f) 0.1 cm; (g–k) 0.2 mm; (l, m) 100 μm; (n–q) 500 μm.

*ad1* mutants are known to display organ fusion defects during both juvenile and male reproductive development (Sinha & Lynch, 1998). To supplement descriptions of previous alleles, we carried out detailed phenotypic analysis of *ad1‐224* plants. Specifically, we observed that in mutant seedlings, the first, second, and occasionally third leaf blades were fused with themselves or with each other, giving rise to a characteristic phenotype in seedlings (Fig. 1a), while at maturity, *ad1‐224* plants showed multiple tassel fusion defects. Normal maize tassels are characterized by several long branches surrounding a central spike; each is covered in grass‐specific structures called spikelets (Fig. 1b). In *ad1* plants, all tassel branches were attached to each other and the central spike (Fig. 1b), while spikelets were fused together and shriveled, with partially emerging anthers (Figs 1c,d, S1). Within each floret, anthers frequently showed fusion as well (Fig. 1e). Longitudinal and transverse sections of young shoots, tassels and spikelets in *ad1* mutants revealed extensive fusion defects. For example, the coleoptile and the first leaf, as well as the first and second leaves, were often fused in *ad1* germinating shoots (Fig. 1h–k), with both the adaxial and abaxial sides of the blade participating in fusion events (Fig. 1i,k), while *ad1* tassel branches and glumes adhered to each other and to the central spike (Fig. 1f,g). Unlike tassels, mature ears of *ad1* mutants did not show a visible phenotype (Fig. S1).

Scanning electron microscope analysis of *ad1* seedling and tassel fusions revealed a range of defects, including misshapen cells surrounding the fusion area in leaves (Fig. 1m), and macrohairs from one surface fused to epidermal cells on another surface (Fig. S1). Areas of fusions between juxtaposed epidermal surfaces were quite extensive and often appeared seamless. Similar extensive and seamless fusions between distinct epidermal cell layers were observed in *ad1* tassel glumes, which were reduced in size, and anthers (Figs 1n–q, S1). We also analyzed developing ear spikelets using SEM and observed fusion events of adjacent glumes (Fig. S1). The seemingly normal appearance of mature ears was likely due to the reduced outgrowth of glumes in ear spikelets when compared to tassel spikelets. Taken together these observations indicated that the organ adherence of *ad1* mutants was caused by epidermal fusions among and between different tissues and organs in both juvenile and reproductive stages, and suggest that AD1 plays an important role in maintaining proper organ separation.

### *AD1* encodes a 3‐KETOACYL‐CoA SYNTHASE involved in cuticular wax biosynthesis

**Fig. 2:**
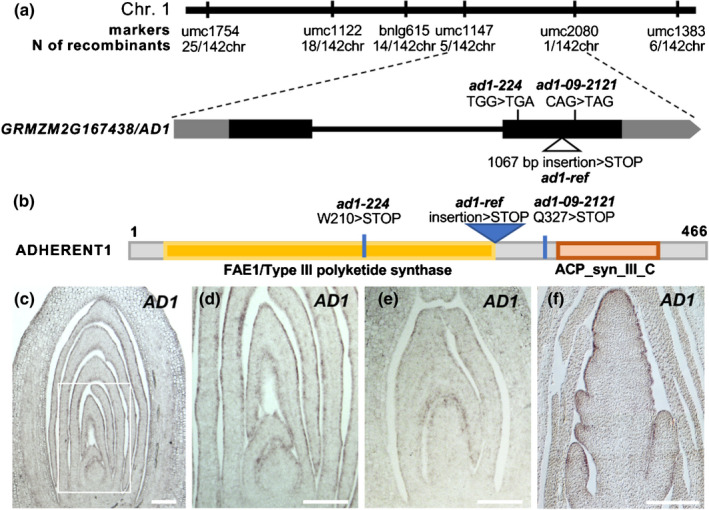
*AD1* encodes a 3‐KETOACYL‐CoA‐SYNTHASE. (a) Positional cloning of *AD1*. Schematic representation of the *AD1* gene and the position of the mutant alleles. Exons are depicted as black rectangles and UTRs are depicted as grey rectangles. (b) Schematic representation of the AD1 protein. ACP_syn_III_C, 3‐Oxoacyl‐[acyl‐carrier‐protein (ACP)] synthase III C terminal (Pfam). (c–f) RNA *in situ* hybridizations with *AD1* antisense probes. (c) Expression pattern in wild‐type maize germinating seedlings, (d) higher magnification of the area framed in (c), (e) shoot region of 20 DAP (day after pollination) embryos, and (f) immature tassel. Bars, 200 μm.

Using an F_2_ segregating population, the *ad1‐224* mutant was mapped to a 10.8 Mb window on chromosome 1 (Fig. 2a). Subsequent bulked segregant whole genome sequence analysis (Dong *et al*., 2019) identified a G to A transition within the coding region of *GRMZM2G167438/Zm00001d032728* (B73v3/v4) in the *ad1‐224* mutant, which introduced a premature stop‐codon (W210 > STOP). *GRMZM2G167438* encodes a member of the 3‐KETOACYL‐CoA SYNTHASE (KCS) family, a group of enzymes necessary for cuticular wax biosynthesis (Yeats & Rose, 2013). To confirm that *GRMZM2G167438* corresponded to *AD1*, we sequenced the candidate gene in three additional alleles (*ad1‐109D*, *ad1‐110E* and *ad1‐09‐2121*). Sequencing results showed that *ad1‐109D* and *ad1‐110E* contained an identical 1067 bp insertion of unknown origin in the second exon, leading to a frame shift (hereafter *ad1‐109D* and *ad1‐110E* are referred to as *ad1‐ref*; Fig. S2), while *ad1‐09‐2121* carried a C to T transition within the second exon that introduced a premature stop‐codon (Fig. 2a,b; Q327 > STOP). Mutations in all three independent alleles were predicted to produce truncated AD1 proteins that disrupted the highly conserved FAE1/Type III polyketide synthase and ACP_syn_III_C domains (Fig. 2b), confirming that knockout of *GRMZM2G167438* caused the adherence phenotype of *ad1* mutants.

Based on quantitative reverse transcription polymerase chain reaction (qRT‐PCR) and available RNA‐seq data, and consistent with the severe phenotype observed in seedlings and tassels, *AD1* showed the highest levels of expression in tassels, ears and seedling leaves (Fig. S2). To characterize the expression pattern of *AD1* in more detail, we carried out RNA *in situ* hybridizations in germinating shoots, embryos and immature tassels. In all tissues tested, *AD1* showed the strongest expression in the epidermal layer (Figs 2c–f, S2). To determine its subcellular localization, we performed confocal co‐imaging of a YFP‐AD1 fusion protein and the ER marker mCHERRY‐CNX1 (Gao *et al*., 2012) in *N. benthamiana* leaf epidermal cells. Strong co‐localization of the YFP‐AD1 and mCHERRY‐CNX1 signals was observed (Fig. S2), indicating that AD1 localized to the ER as expected (Haslam & Kunst, 2013). Altogether, the expression of *AD1* in epidermal cells and the subcellular localization of AD1 in the ER are consistent with a role for AD1 in cuticular wax biosynthesis (Yeats & Rose, 2013).

Individual members of the KCS family have diverse roles throughout the plant, functioning in seed oil production, suberin metabolism, and cuticle lipid formation (James *et al*., 1995; Todd *et al*., 1999; Yephremov *et al*., 1999; Pruitt *et al*., 2000; Franke *et al*., 2009; Lee *et al*., 2009; Kim *et al*., 2013). *KCS* genes often show distinct substrate specificity, although the biological role of most *KCS* genes remain largely unknown (Haslam & Kunst, 2013). Previous phylogenetic analysis revealed eight evolutionarily conserved subclasses of *KCS* genes, including 21 from Arabidopsis and 26 from maize (Joubes *et al*., 2008; Campbell *et al*., 2019), and we included two additional genes present in the maize B73v3 reference genome. AD1/ZmKCS19 lies within the θ subclass that contains three uncharacterized Arabidopsis *KCS* genes, and four additional maize homologs (Fig. S3). The θ subclass is evolutionarily conserved but of unknown function, and shows distinct sequence features relative to the other subclasses, suggesting it may have unique activity. Interestingly, FDH/AtKCS10, whose mutants also show organ fusions in Arabidopsis (Yephremov *et al*., 1999; Pruitt *et al*., 2000), belongs to a separate distant clade from AD1/ZmKCS19 (Fig. S3).

To understand why *ad1* single mutants showed a severe phenotype in maize despite the presence of four homologs (*ZmKCS3*, *ZmKCS24*, *ZmKCS25*, *ZmKCS28*), we analyzed the expression of *AD1* in various tissues relative to other *KCS* genes using published RNA‐seq data (Stelpflug *et al*., 2016). *AD1* expression was higher than other θ subclass members in most tissues, with the exception of the pericarp. In tissues where *ad1* displayed strong phenotypes (i.e. seedling leaves and tassels), very little expression from other genes in the θ subclass was apparent (Fig. S2). These results suggest that tissue‐specific expression of *AD1* likely explains why loss of *AD1* function cannot be compensated by closely related family members. We also compared *AD1* expression patterns to the maize *KCS* genes from other subclasses and observed that many were broadly expressed in various tissues similar to *AD1* (Fig. S2). Taken together, these results suggest that loss of *AD1* function cannot be buffered by family members from other subclasses, despite showing largely overlapping expression patterns (i.e. *GRMZM2G162508/ZmKCS8*), and support an independent function for the θ subclass of KCS enzymes.

### Cuticular wax biosynthesis and deposition are defective in *ad1* mutants

**Fig. 3:**
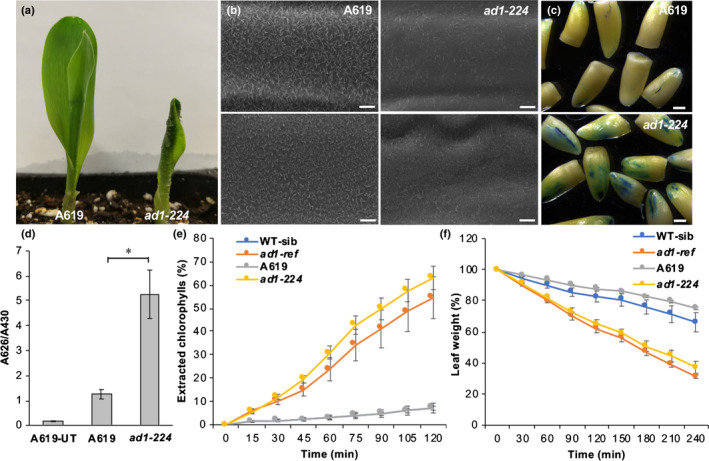
Epicuticular wax crystals and transpiration analysis. (a) Leaves of wild‐type maize A619 and *ad1‐224* seedlings misted with water. (b) Scanning electron microscope images of epicuticular wax crystals on the third leaf sheath (top) and the third leaf lamina (bottom) in wild‐type and *ad1‐224* mutants. Bars, 5 μm. (c) Toluidine blue staining of etiolated coleoptiles (excised post‐staining). Bars, 1 mm. (d) Quantification of toluidine blue uptake in the coleoptile of young seedlings, normalized to Chl content. *n* = 5 (five seedlings per repetition; Student's *t*‐test: *, *P* < 0.001). Error bars represent SD. UT, untreated control. (e) Chlorophyll leaching assays showing extracted Chl at individual time points. *Y*‐axis values indicate percentage relative to that at 24 h after initial immersion (note that WT‐sibling and A619 data tightly overlap). Error bars show SD, *n* = 3. (f) Water loss assays. Error bars show SD, *n* = 3. *Y*‐axis values indicate percentage relative to the leaf weight at time 0.

To investigate whether loss of AD1 function influenced cuticular wax deposition, we tested the ‘lotus effect’, a leaf self‐cleaning mechanism of many plants that has been correlated with the abundance of epicuticular wax crystals (Barthlott & Neinhuis, 1997; Zheng *et al*., 2019). Wild‐type and *ad1* mutant seedlings were misted with water, and water droplets accumulated only on the surface of *ad1* leaves (Fig. 3a), suggesting epicuticular wax crystal defects. We therefore analyzed the deposition of epicuticular wax crystals on the surface of young leaves by SEM. Strikingly, the number and size of wax crystals were considerably reduced in *ad1* mutant leaves relative to wild‐type (Figs 3b, S4).

To understand whether these wax defects affected cuticle permeability, we quantified the accumulation of toluidine blue stain in coleoptiles. We detected a higher accumulation of the dye in *ad1* mutants relative to wild‐type, suggesting increased cuticle permeability during embryogenesis (Fig. 3c,d). In addition, cuticular transpiration, measured as loss of leaf weight, and Chl extraction occurred more rapidly in *ad1* mutant alleles relative to wild‐type (Fig. 3e,f), further confirming defects in cuticular wax accumulation of *ad1* mutants.

**Fig. 4:**
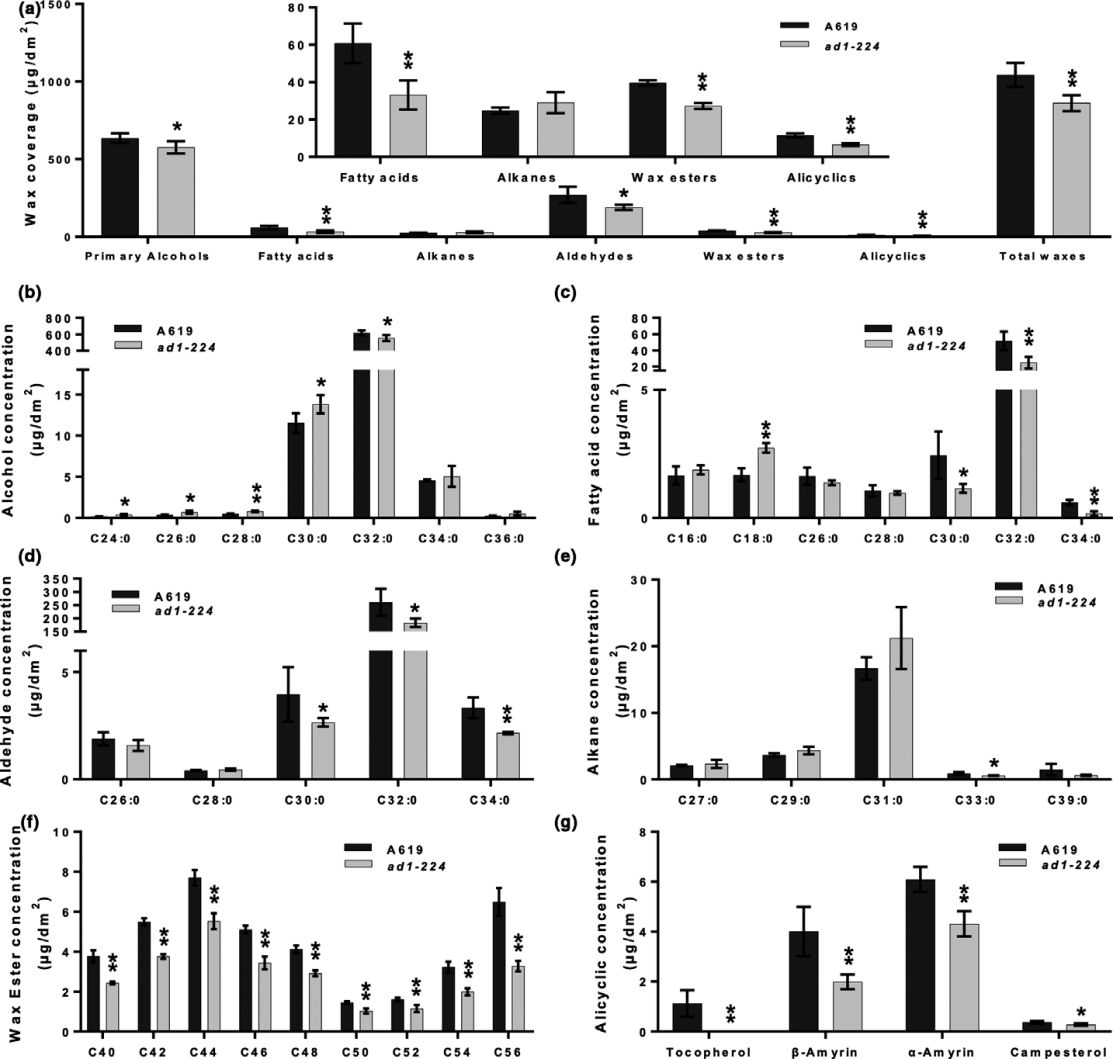
*ad1* cuticular wax analysis. (a) Total wax coverage and amount of each wax class in *ad1‐224* mutants and wild‐type maize A619 leaves. The inset shows the less abundant wax classes at a different scale to more clearly visualize significant differences. (b–g) Concentration of individual components in each wax class: primary alcohol (b), fatty acid (c), aldehyde (d), alkane (e), wax ester (f) and alicyclic (g). Means of four replicates and SD are reported. *, *P* < 0.05; **, *P* < 0.01, according to Student's two‐tailed *t*‐test.

The reduced amount of epicuticular wax crystals on the surface of *ad1* leaves together with increased permeability suggested that *AD1* is involved in cuticular wax biosynthesis. Therefore, we evaluated the amount and composition of cuticular waxes in the third leaves of wild‐type and *ad1* plants using GC‐MS and GC–FID (Figs 4, S4). The amount of total wax was significantly lower in *ad1‐224* (*c.* 18%) and *ad1‐ref* (*c.* 23%) than in wild‐type leaves (Figs 4a, S4). Maize leaf cuticular waxes are composed of a mixture of compounds, including VLCFAs, alkanes, alcohols, aldehydes, ketones, wax esters and alicyclic compounds (Bourgault *et al*., 2020). All components were significantly decreased in *ad1‐224* compared with wild‐type, with the exception of alkanes (Fig. 4a). Similar results were obtained with the *ad1‐ref* allele for most compounds, although primary alcohols and fatty acids showed no significant difference from wild‐type concentrations (Fig. S4). The primary alcohol fraction was the most abundant class of juvenile leaf waxes (Figs 4a, S4), with the C_32:0_ primary alcohol being the dominant homolog, as expected (Bianchi *et al*., 1978). In fact, this single component (C_32:0_ primary alcohol) constituted over 60% of the overall wax load in the samples studied. The amount of C_32:0_ primary alcohol in *ad1‐224* and *ad1‐ref* mutants was decreased by *c.* 11% and 13% respectively, relative to the wild‐type (Figs 4b, S4). Another abundant component was C_32:0_ aldehyde, which constituted *c.* 25% of the total wax load. In *ad1‐224* and *ad1‐ref* mutants, the C_32:0_ aldehyde load was reduced by *c.* 30% and 48%, respectively, compared with values in wild‐type samples (Figs 4d, S4). Less abundant components, including C_30:0_–C_34:0_ fatty acids, C_30:0_ and C_34:0_ aldehydes, C_33:0_ alkane and all four identified alicyclic compounds, showed lower concentrations in *ad1‐224* mutants than in control samples (Fig. 4c,e,g). In *ad1‐ref* mutants, leaf waxes showed reduced amounts of C_34:0_ fatty acid, C_28:0_–C_34:0_ aldehydes, C_33:0_ and C_39:0_ alkanes, tocopherol and campesterol (Fig. S4). All components of the wax ester fraction, which corresponds to less than 5% of the overall wax load, were decreased in both alleles of *ad1* relative to wild‐type (Figs 4f, S4; Dataset S1). Altogether, *ad1* mutants had reduced amounts of alkanes and primary alcohols with chains longer than 31 carbons, whereas aldehydes and fatty acids were affected in components longer than 28 carbons. The fact that wax esters showed significantly reduced loads in both mutant alleles indicates that at least one of their major individual components, acyl and alkyl chains, were also reduced in the mutants. For example, the C_46_ wax ester homolog group contains mostly 20:0–22:0 acyl chains and 24:0–26:0 alkyl chains (Bourgault *et al*., 2020), suggesting that components with chain lengths shorter than 28 carbons were also affected. Thus, the chemical data does not clearly reflect any trends in terms of the acyl chain length specificity of AD1.

### The *fused leaves1* mutant enhances the cuticle defects of *ad1*

**Fig. 5:**
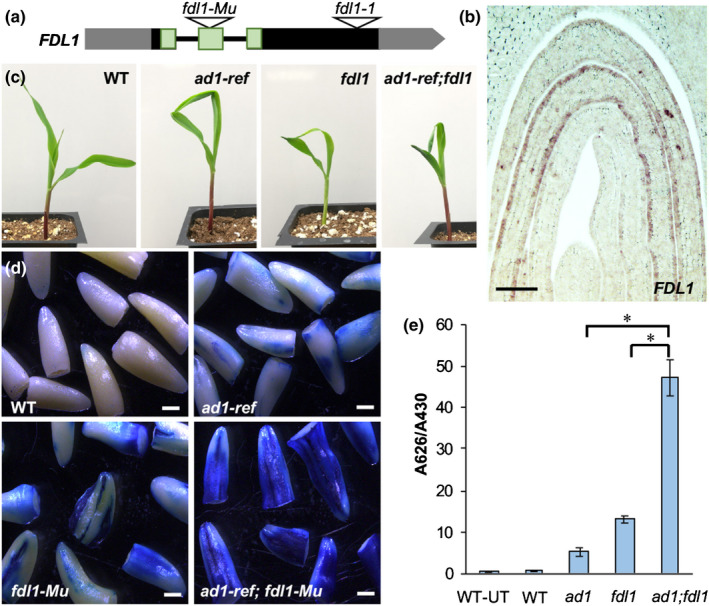
*FDL1* regulates cuticle formation. (a) Schematic representation of the maize *FDL1* gene showing the transposon insertion in the second exon (black and grey rectangles, exons and UTR regions, respectively). The predicted DNA binding domain is shaded in green. The position of the previously characterized *Spm* insertion in the *fdl1‐1* allele is also shown. (b) RNA *in situ* hybridization with an *FDL1* antisense probe in germinating seedlings. Bar, 100 µm. (c) The seedling phenotype of wild‐type, *ad1‐ref*, *fdl1‐Mu* and *ad1‐ref;fdl1‐Mu*. (d) Toluidine blue assay in etiolated coleoptiles excised post‐staining. Bars, 1 mm. (e) Quantification of toluidine blue uptake by the coleoptile of young seedlings, normalized to Chl content. *n* = 5 (five seedlings per repetition; *, *P* < 0.001, according to Student's *t*‐test). Error bars represent SD; UT, untreated samples.

The previously characterized maize mutant *fdl1* has a phenotype similar to *ad1,* showing seedling leaf fusion and cuticular wax defects. *FDL1* (*GRMZM2G056407/Zm00001d022227*) encodes ZmMYB94, an R2R3 MYB TF whose closest Arabidopsis homolog regulates cuticular wax biosynthesis genes (Raffaele *et al*., 2008; Seo *et al*., 2011; La Rocca *et al*., 2015). However, Arabidopsis *myb94* mutants do not show organ fusion defects, suggesting differences between species. Characterization of a newly obtained transposon insertion allele that disrupts the FDL1 DNA binding domain (*fdl1‐Mu*; Fig. 5a), showed that homozygous *fdl1‐Mu* mutants displayed seedling leaf fusions (Fig. 5c), slightly reduced epicuticular wax crystals, increased nonstomatal water loss, and faster Chl leaching relative to wild‐type plants (Fig. S5), similar to the previously reported *fdl1‐1* allele (La Rocca *et al*., 2015). These phenotypes strongly resembled those of *ad1* (Fig. 3), with the exception that no tassel defects were observed in *fdl1‐Mu* mutants. RNA *in situ* hybridizations showed that *FDL1* was strongly expressed in the epidermal layer of young leaves and tassels, in a pattern remarkably similar to *AD1* (Figs 5b, S5). Overall, these data suggest that *AD1* and *FDL1* may function in the same pathway.

To explore this possibility, we generated *ad1;fdl1‐Mu* double mutants. Homozygous *ad1;fdl1‐Mu* double mutants showed the characteristic leaf fusion phenotype seen in both single mutants (Fig. 5c), while SEMs of the leaf surface showed that the epicuticular wax deposition defects of *ad1‐ref* were enhanced by the *fdl1* mutation (Fig. S6). Accordingly, water loss by cuticular transpiration and Chl leaching occurred more rapidly in *ad1‐ref;fdl1‐Mu* double mutants compared to each single mutant and wild‐type (Fig. S6). The accumulation of toluidine blue stain in coleoptiles was also higher in *ad1;fdl1* double mutants relative to single mutants (Fig. 5d,e). Double mutant adult plants also displayed a higher frequency of leaf fusion events relative to *ad1* and appeared more sensitive to drought stress (Fig. S6). These results highlight the importance of both genes for resistance to environmental stresses and their synergistic interaction.

### FDL1 directly binds regulatory regions of *AD1* and many additional genes involved in cuticle formation

**Fig. 6:**
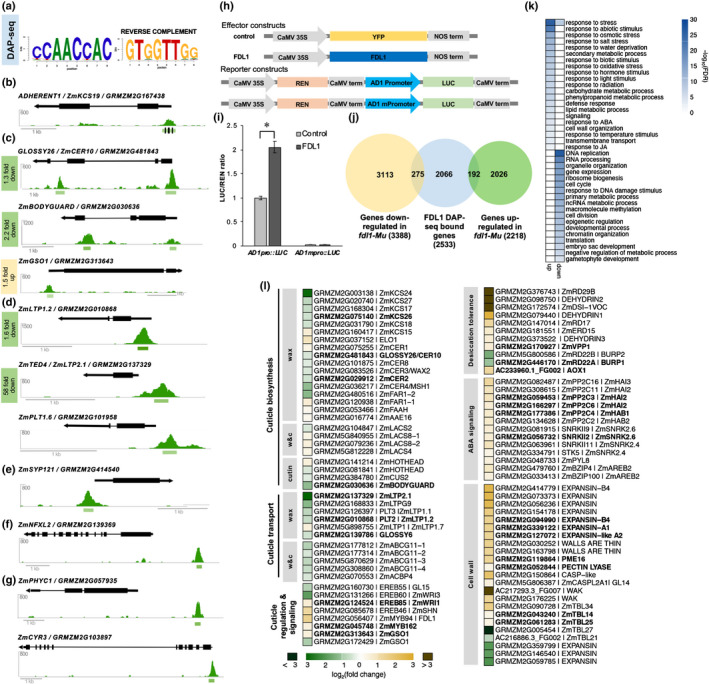
Direct targets of FDL1 function. Genome wide binding and differential expression analysis of FDL1/ZmMYB94 in maize. (a) Top enriched motif present in FDL1 bound regions identified by DAP‐seq. (b) The 5′UTR of *AD1/KCS19* is directly bound by FDL1. Green bars represent 200 bp peak. Black lines show location of CCAACC motifs tested by mutational analysis. (c) Examples of additional cuticular wax and cutin related genes that are directly bound by FDL1. (d) FDL1 directly binds several *LIPID TRANSFER PROTEINS* (*LTPs*) genes putatively involved in secretion of cuticular wax. (e) FDL1 directly binds *SYNTAXIN 121*, encoding a SNARE complex protein involved in membrane trafficking and osmotic homeostasis, previously not linked to cuticle formation. (f) FDL1 directly binds *ZmNFXL2*, an NF‐X transcription factor recently associated with cuticle defects. (g) Several light‐regulated genes (*ZmPHYTOCHROME C*, *ZmCRYPTOCHROME 3*) are directly bound by FDL1, suggesting a link between cuticle formation and light. (h) Schematic diagram of effector and reporter constructs used for transcriptional activation assay. (i) *AD1* transcriptional activation by FDL1. Error bars show SD, *n* = 4. *, *P* < 0.001, according to Student's *t*‐test. (j) Overlap of genes directly bound by FDL1 in DAP‐seq and genes differentially expressed in *fdl1‐Mu* RNA‐seq in 3‐d old coleoptiles. (k) GO enrichment analysis of upregulated and downregulated genes in *fdl1‐Mu* coleoptiles. (l) Differentially expressed genes in *fdl1‐Mu* coleoptiles involved in cuticle formation and other processes. Bold font indicates genes directly bound by FDL1 in DAP‐seq. w&c, wax and cutin.

To test whether *AD1* was among the target genes directly regulated by FDL1, we performed DAP‐seq, an *in vitro* DNA‐TF binding assay that captures genomic DNA binding events in their native sequence context (O'Malley *et al*., 2016; Galli *et al*., 2018). FDL1 protein was incubated with a maize genomic DNA library, and FDL1‐bound DNA fragments were identified using next‐generation sequencing. In total, 10 028 peaks were detected, of which 2737 (27%) were located near genes (defined as 10Kb upstream of the TSS to 3Kb downstream of the TTS) (Fig. S7). These peaks targeted 2533 unique genes and preferentially bound to sequences containing a CCAACCAC motif (Fig. 6a; Dataset S2). This consensus motif was similar to the motif identified in DAP‐seq experiments for the homologous AtMYB94 and AtMYB96 (Fig. S7) (O'Malley *et al*., 2016). It also resembled that reported in the promoters of several cuticle‐related target genes of AtMYB94 and AtMYB96, although several nucleotides outside of the core AAC motif were divergent in these genes, suggesting species‐specific motif preferences (Seo *et al*., 2011; Lee & Suh, 2015b).

Among the genes bound by FDL1, *AD1* showed a binding peak in the 5’UTR, which harbored four CCAACC motifs (Fig. 6b), conserved across three different maize inbred lines (Fig. S7). An EMSA with FDL1 protein and a 280bp *AD1* 5′UTR‐labeled probe confirmed the binding specificity of this motif, which was abolished when three of the four CCAACC motifs were mutated (Fig. S7). These results support the finding that FDL1 binds to the core CCAACC sequence. Because *AD1* was directly bound by FDL1 and the *fdl1* leaf fusion defects originate during embryo development (La Rocca *et al*., 2015; Castorina *et al*., 2020), we performed qRT‐PCR in *fdl1‐Mu* embryos and found that *AD1* was downregulated (Fig. S7). This suggested that FDL1 activated expression of *AD1*. To test this, we performed a trans‐activation assay in maize leaf protoplasts using a *35S::FDL1* effector construct and luciferase reporter construct containing 1.6kb of upstream *AD1* sequence. We observed a two‐fold increase in luciferase activity that was dependent on the CCAACC motifs because mutation of these motifs abolished transcriptional activity (Fig. 6h,i). Collectively, these results suggest that FDL1 is a positive regulator of *AD1* during embryogenesis.

In addition to *AD1*, many other genes involved in cuticle formation were also directly bound by FDL1 (Figs 6c, S8; Table S2). These included cutin and cuticular wax biosynthesis candidate genes such as *GLOSSY26/ZmCER10/ZmECR*, *ZmBODYGUARD1* (*ZmBDG1*), *GLOSSY8A/ZmKCR*, *GLOSSY2/ZmCER2*, as well as several additional *KCS* genes. Several cutin and wax transporters including the *ZmABCG11* family and *GLOSSY6* were also directly bound (Li *et al*., 2019). In addition, the 5’UTR of the putative maize co‐ortholog of the Arabidopsis receptor‐like kinase GSO1, which functions in cuticle surveillance and reinforcement (Doll *et al*., 2020) was directly bound by FDL1 (Fig. 6c) as were fatty acid‐related enzymes (i.e. ZmHAD/GRMZM2G055667) and several WRINKLED‐type AP2 transcription factors (TFs) that regulate cutin and fatty acid synthesis (Li‐Beisson *et al*., 2013). Of the cuticle‐related maize genes bound by FDL1, the Arabidopsis orthologs of *ZmKCS26* (*AtKCS2/ZmDAISY*), *GLOSSY2* (*AtCER2*) and *GLOSSY26* (*AtCER10*) have been shown to be bound by AtMYB94, the closest FDL1 Arabidopsis homolog (Lee & Suh, 2015b), suggesting that despite differences in motif binding, certain targets are conserved among MYB94‐type TFs.

Some of the strongest peaks in our FDL1 binding dataset were in the proximal regulatory regions of several *LIPID TRANSFER PROTEINS* (*LTP*) genes (Fig. 6d), hypothesized to export waxes from epidermal cells to the extracellular space (Wei & Zhong, 2014). Interestingly, the promoter of *ZmLTP1.6* is used in the maize BBM/WUS transformation system to drive highly cell‐type specific *WUS* expression and induce somatic embryogenesis (Jones *et al*., 2019). The strong binding of FDL1 to both *ZmLTP1.6* and its close homolog *ZmLTP1.2* suggests that FDL1 is a key component of this promoter cassette and could guide alternative synthetic promoters. Also, these results strongly suggest that these LTPs are major players in maize epicuticular wax export.

Several other genes associated with lipid‐related processes or suspected roles in cuticle formation, were also strongly bound by FDL1. These included genes encoding a phosphatidylserine synthase, ZmPSS1/GRMZM2G039385, whose Arabidopsis homolog functions in phospholipid synthesis in pollen and meristem development (Liu *et al*., 2013), ZmSYP121/GRMZM2G414540, a syntaxin that is involved in vesicle trafficking in response to drought, abscisic acid (ABA), and pathogen attack (Karnik *et al*., 2015), as well as ZmNFXL2/GRMZM2G139369, a stress‐responsive TF associate with modified cuticle properties (Lisso *et al*., 2012). Light has also recently been reported to have a role in cuticle development (Castorina *et al*., 2020; Qiao *et al*., 2020). FDL1 could facilitate this process via binding to *PHYTOCHROME C1* and *CRYPTOCHROME3*, which mediate red and blue light‐dependent transcriptional changes, respectively (Fig. 6g; Table S2). Overall, genome‐wide FDL1 binding data greatly expands the number of putative target genes of MYB94‐type TFs, offering unique insights into cuticle formation that are likely applicable across species.

### Loss of FDL1 function results in large transcriptional reprogramming during seedling development

The DAP‐seq technique captures global DNA‐binding events across all tissues and conditions. To focus on transcriptional programs regulated by FDL1 during early development, we performed RNA‐seq on 3‐d old coleoptiles from *fdl1‐Mu* mutant and wild‐type seedlings. We identified 5606 differentially expressed genes (DEGs), including 3388 upregulated and 2218 downregulated genes compared with wild‐type (adjusted *P*‐value < 0.05) (Fig. 6j; Dataset S3), indicating that loss of FDL1 results in large transcriptional reprogramming early in development. Of the 5606 DEGs, 467 were bound by FDL1. These included 192 downregulated and 275 upregulated genes (Dataset S4), suggesting that FDL1 may act as either an activator or repressor, possibly depending on interacting co‐factors.

Gene ontology (GO) enrichment analysis of all upregulated genes showed strong enrichment for environmental and stress responses including defense, osmotic stress, salt stress, water deprivation, temperature stimulus, UV, and wounding (Fig. 6k). In particular, drought‐induced genes such as *DEHYDRINS* and *RESPONSIVE TO DESICCATION* (*RD*) genes were strongly upregulated, as well as genes involved in lipid and phenylpropanoid metabolism, and cell wall organization (Fig. 6l). These included *R1/ZmBHLH1*, a master regulator of maize anthocyanins (5.5‐fold upregulated), along with several enzymes involved in flavonoid and lignin biosynthesis, such as *PAL*, *C4H*, *4CL*, and *HCT* (Fig. S9). In addition to their role in combating various stresses, these compounds are also minor constituents of cutin (Lee & Suh, 2015a; Bourgault *et al*., 2020). Additional upregulated genes whose Arabidopsis homologs are associated with cutin and cuticular wax included several *LACS* cutin biosynthesis genes, *ZmCER1*, *ZmHOTHEAD*, and *ZmELO1* (Fig. 6l) (Yeats & Rose, 2013). Abscisic acid affects both cuticle composition and drought response (Martin *et al*., 2017). Accordingly, upregulated genes associated with the ‘ABA‐response’ GO term included several key ABA signal transduction pathway components, many of which were also bound by FDL1 (*PP2C3*/*ZmHAI2*, *PP2C6/ZmHAI2*, *PP2C4/ZmHAB1* and the maize *SNRK2.6* homolog *SNRKII2*; Fig. 6l; Table S2). These results suggest that FDL1 may directly mediate ABA signaling. Of the upregulated genes in the cell wall‐related GO category, we observed lignin biosynthesis, EXPANSIN, PECTIN LYASE, WALL‐ASSOCIATED KINASE (WAK), CASP‐like cell wall modifying enzymes, and cellulose biosynthesis *TBL* genes, signifying possible coordination between leaf cuticle development and cell wall biosynthesis (Fig. 6l).

While direct binding of FDL1 was observed in the regulatory regions of several of these genes (i.e. *ZmRD22*, *PP2C/ZmHAIs* and *EXPANSINs*; Fig. S8), for many there was no evidence of direct regulation by FDL1. We therefore hypothesize that the increased transpiration in the *fdl1‐Mu* mutant activated compensatory or protective pathways that do not require FDL1. These potentially include *ZmEREB46*, a homolog of AtWIN/SHN known to directly activate several cuticle‐related genes in Arabidopsis (2.4‐fold upregulated in *fdl1‐Mu* mutants), and *MYB162*, a closely related homolog of *FDL1*, upregulated and directly bound by FDL1 (Fig. 6l; Table S2). This suggests a signaling network with many compensatory mechanisms to safeguard the critical process of cuticle development.

In contrast to the GO terms associated with upregulated genes, *fdl1‐Mu* downregulated genes were most strongly enriched for terms related to DNA and primary metabolism, RNA processing, cell cycle, gene expression, and epigenetic regulation (Fig. 6l). Many of these processes play fundamental roles in fast developing tissues and their downregulation suggests a metabolic slow‐down in *fdl1‐Mu* coleoptiles. Overall, downregulation of cell growth related processes together with the upregulation of stress‐related genes may indicate that defects in cuticle formation trigger a metabolic shift that favors tissue protection over growth.

Notably, downregulated genes also included many with well‐established roles in cutin and cuticular wax‐related processes such as *ZmBDG1*, *LTPs* and other lipid transporter genes, *KCS* genes, and *GLOSSY6* and *ZmCASPL2A1*, whose role in cuticle formation is still undefined (Li *et al*., 2019; Zheng *et al*., 2019) (Fig. 6l). The regulatory regions of several of these were also directly bound by FDL1 (Figs 6c,l, S8). Among the *KCS* genes, we did not detect any differential expression of *AD1* in our coleoptile RNA‐seq dataset despite the observed direct binding of FDL1 in DAP‐seq and EMSA, and our qRT‐PCR results showing downregulation in embryos (Figs 6b, S7). This suggests potential activation by other TFs in *fdl1‐Mu* coleoptiles (possibly other closely related co‐expressed MYBs; Fig. S9). Interestingly, Zm*KCS24/GRMZM2G003138*, a closely related homolog of *AD1* from the θ subclass was strongly downregulated (7‐fold; Fig. 6l) but not directly bound by FDL1, highlighting the distinct regulation of certain members of this subclass. Other notable downregulated genes included a homolog of *AtWRINKLED3* (*GRMZM2G131266/EREB60*) (To *et al*., 2012), and *GLOSSY15*, which regulates several juvenile leaf characteristics related to epicuticular waxes (Lauter *et al*., 2005). In total, manual curation identified 25 downregulated and 18 upregulated genes associated with cuticle formation (Fig. 6l). In several cases, related family members showed opposing differential regulation (i.e. *ZmKCS15/GRMZM2G160417* was upregulated while five other *KCS* genes were all downregulated). In general, genes involved in cuticular wax biosynthesis and transporters tended to be downregulated in the *fdl1‐Mu* mutant (Fig. 6l).

Interestingly, two of the differentially expressed genes that were directly bound by FDL1 corresponded to GWAS hits for water use efficiency. These included *ZmVPP1,* which encodes an INORGANIC H PYROPHOSPHATASE whose enhanced expression and drought tolerance is associated with MYB binding site recruitment (Wang *et al*., 2016), and a SEC14 homolog (*GRMZM2G704053/Zm00001d033836*) involved in intracellular lipid trafficking (Lin *et al*., 2020). Taken together, these results indicate that FDL1 regulates numerous pathways and could be exploited as a means of increasing water use efficiency in maize.

## Discussion

Cuticle formation is essential for organ separation in plants. Of the many genes with known cuticle defects however, relatively few show organ fusions when mutated (Ingram & Nawrath, 2017). The fusion events observed in *ad1* mutants show that AD1/ZmKCS19, a cuticular wax biosynthesis enzyme, is necessary for organ separation throughout maize development. KCS enzymes catalyze the rate limiting step in the production of VLCFA precursors of cuticular waxes and determine their length (Millar & Kunst, 1997). When FAE1, a seed‐specific KCS, is ectopically expressed in leaves, the transgenic leaves increase the production of VLCFAs that mirror those found in the seeds. Similarly, loss of CER6 or KCS1 function results in accumulation deficiencies in the biosynthesis of C26 and longer. However, mutants of the KCS‐encoding *FDH* gene in Arabidopsis and its rice homolog *ONION1* (*ONI1*) result in dwarf plants and organ fusions (Yephremov *et al*., 1999; Efremova *et al*., 2004; Ito *et al*., 2011). Whereas *fdh* rosette leaves produce increased amounts of cutin and waxes compared to wild‐type plants (Yephremov *et al*., 1999; Voisin *et al*., 2009), *oni1* had a reduced amount of VLCFAs without a clear chain‐length specificity (Ito *et al*., 2011). Unlike the *fdh* mutants and similarly to *oni1* seedlings, *ad1* seedlings did present reductions in certain very long chain wax components, but those changes were not consistent in terms of the affected chain lengths of different wax classes. Thus, it could be speculated that AD1 is involved in producing very long chain signaling lipids with roles in epidermal cell adhesion, similar to FDH (Pruitt *et al*., 2000).

Genome‐wide DAP‐seq and expression analysis indicated that FDL1 directly binds to regulatory regions of many cuticle‐related biosynthesis and transport genes, including *AD1*. Accordingly, many of these genes were downregulated in *fdl1* coleoptiles, indicating that in the early stages of germination, FDL1 is crucial for cuticle formation, and that FDL1 activates their transcription. Interestingly, FDL1 binds to *ZmBDG1*, whose Arabidopsis homolog is required for cutin synthesis, and *ZmBDG1* was downregulated in coleoptiles, suggesting that FDL1 might promote cutin biosynthesis. Both cutin and wax loads were indeed reportedly reduced in *fdl1‐1* coleoptiles (Castorina *et al*., 2020).

Among targets bound by FDL1 was *AD1* itself. This direct regulation is likely crucial early in development, given that we observed *AD1* downregulation in *fdl1* embryos (qRT‐PCR) but not in coleoptiles (RNA‐seq), and that the cuticle composition of *fdl1* plants is predominantly affected in the first embryonically initiated leaves (Castorina *et al*., 2020). It is likely that other MYB TFs function redundantly with FDL1 in later stages of development. For example, we identified eight *MYB* genes that were upregulated in *fdl1* coleoptiles that could provide genetic buffering (Rodriguez‐Leal *et al*., 2019). Intriguingly, the regulatory regions of some of these genes were bound by FDL1, suggesting they may be directly repressed by FDL1 in certain conditions.

In Arabidopsis, several MYBs are known to play a role in cuticle formation (i.e. AtMYB30, AtMYB94 and AtMYB96). AtMYB94 and AtMYB96 are the most closely related to FDL1 and have been shown by EMSA and chromatin immunoprecipitation (ChIP)‐PCR to directly regulate cuticle‐related genes (Seo *et al*., 2011; Oshima *et al*., 2013; Lee & Suh, 2015b). The maize orthologs of several of these genes were directly bound by FDL1 in our DAP‐seq dataset (Table S2). However, the motifs necessary for binding in maize (CCAACC) appear to differ slightly from those reported for these Arabidopsis genes. Therefore, while a core regulatory module composed of MYB TFs and cuticle‐related genes appears conserved between Arabidopsis and maize, many species‐specific features exist. Furthermore, our unbiased genome‐wide binding approach revealed that FDL1 directly binds many genes for which no direct connection with cuticle formation has been described. These include secretory system components, phospholipid signaling genes, fatty acid biosynthesis precursors, and cell wall modifying genes (Figs 6, S8; Table S2). In addition, our data suggest that FDL1 also participates in various aspects of stress response regulation (drought, ABA, reactive oxygen species (ROS) and flavonoid production among others). Consistent with these findings, our drought treatment results showed that loss of function of *FDL1* enhanced the sensitivity of *ad1* to water loss. Furthermore, At*MYB30/94/96* are induced by drought and pathogens (Raffaele *et al*., 2008; Seo *et al*., 2011; Lee & Suh, 2015a), suggesting that the many stress‐related target genes of FDL1 identified here might be conserved in other species and could be used to devise strategies to promote stress tolerance in crops.

## Author contributions

XL, JS and AG conceived the study; XL, JS, RB, ZC, MG, JD and AG performed all experiments; XL, RB, MG, FF, JD, IM and AG analyzed data; XL, MG and AG wrote the manuscript with input from all authors. RB and MG contributed equally to this work.

## Supporting information


**Dataset S1** Wax analysis of *ad1* mutant leaves (raw data).

**Dataset S2** List of genes bound by FDL1 (DAP‐seq).

**Dataset S3** List of differentially expressed genes in *fdl1* mutants (RNA‐seq).

**Dataset S4** List of differentially expressed genes bound by FDL1.

**Fig. S1** Phenotypic characterization of *ad1* alleles.
**Fig. S2** Expression analysis of the *KCS* gene family in maize.
**Fig. S3** Neighbor‐joining phylogenetic tree.
**Fig. S4** Cuticular wax analysis in *ad1‐ref* mutants.
**Fig. S5** Analysis of *FDL1* function.
**Fig. S6** Double *ad1‐ref;fdl1‐Mu* mutant analysis.
**Fig. S7** FDL1 DNA binding analysis.
**Fig. S8** Genome browser screenshots of FDL1 binding.
**Fig. S9** Analysis of *FDL1* expression and binding targets.
**Table S1** List of primers used in this study.
**Table S2** List of genes involved in cuticle formation directly bound by FDL1 in DAP‐seq.
Please note: Wiley Blackwell are not responsible for the content or functionality of any Supporting Information supplied by the authors. Any queries (other than missing material) should be directed to the *New Phytologist* Central Office.

## Data Availability

The sequence of *AD1* was deposited in GenBank (accession no. MN872839) and corresponds to GRMZM2G167438_T01/Zm00001d032728_T001 (B73v3/v4). The FDL1 DAP‐seq and RNA‐seq datasets were deposited in the Gene Expression Omnibus (GEO) database (accessions nos. GSE142847 and 149577).
