# Supplementary material for: The FUSED LEAVES1‐ADHERENT1 regulatory module is required for maize cuticle development and organ separation
Source: New Phytol. 2020 Aug 27;229(1):388–402. doi: 10.1111/nph.16837 (PMC7754373; doi:10.1111/nph.16837)
Supplement: Supplementary file 1 — Dataset S1 Wax analysis of ad1 mutant leaves (raw data). [file NPH-229-388-s001.pdf]

SUPPORTING INFORMATION

THE FUSED LEAVES1-*ADHERENT1* REGULATORY MODULE IS REQUIRED FOR MAIZE CUTICLE DEVELOPMENT AND ORGAN SEPARATION

Xue Liu, Richard Bourgault, Mary Galli, Josh Strable, Zongliang Chen, Fan Feng, Jiaqiang Dong, Isabel Molina, Andrea Gallavotti

Article acceptance date: 22 July, 2020.

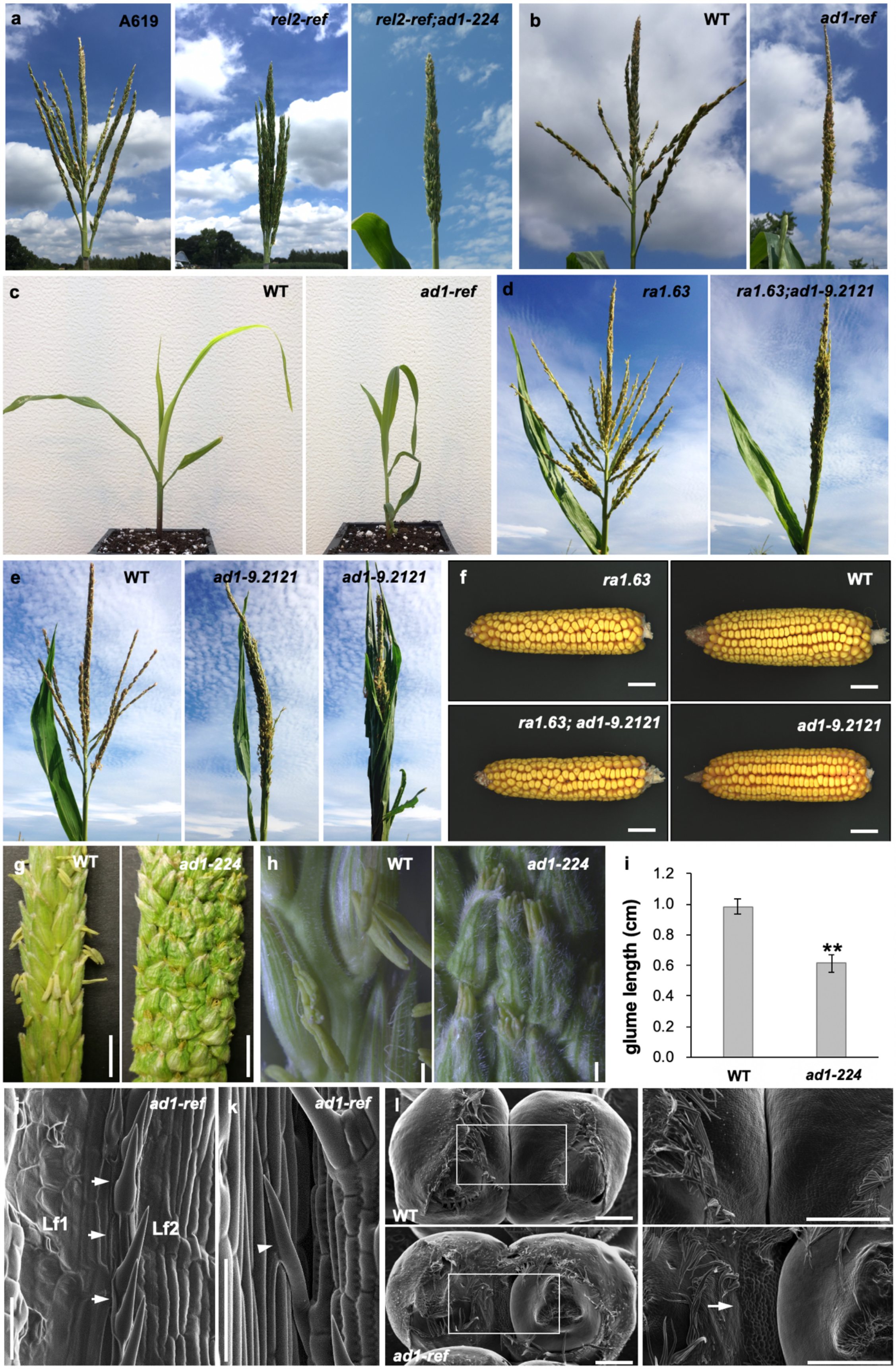
a
A619
rel2-ref
rel2-ref;ad1-224
b
WT
ad1-ref
c
WT
ad1-ref
d
ra1.63
ra1.63;ad1-9.2121
e
WT
ad1-9.2121
ad1-9.2121
f
ra1.63
WT
ra1.63; ad1-9.2121
ad1-9.2121
g
WT
ad1-224
h
WT
ad1-224
i
glume length (cm)
1.2
1.0
0.8
0.6
0.4
0.2
0.0
**
WT
ad1-224
j
ad1-ref
Lf1
Lf2
k
ad1-ref
l
WT
ad1-ref

Figure S1. Phenotypic characterization of *ad1* alleles.

(a) The tassel phenotype of A619, *rel2-ref* and *rel2-ref;ad1-224* original M2 family. (b) The tassel phenotype of wild type sibling and *ad1-ref* mutants. (c) The seedling phenotype of wild type sibling and *ad1-ref* mutants. (d) The original *ad1-9.2121* allele in *ra1-63.3359* Mo17 background. (e) The *ad1-9.2121* allele in BC3(B73). (f) Mature ear phenotype of *ad1-9.2121* mutants. No visible defects is observed. (g) Tassel spikelets of wild type and *ad1-224* mutants. Scale bars, 1 cm. (h) Images of glumes in wild type and *ad1-224* mutant tassels. Scale bars, 0.1 cm. (i) Quantification of the glume length of wild type and *ad1-224* mutants ($n \geq 20$). Error bars show SD; $^{**}p<0.001$. (j) SEM image showing the second leaf (Lf2) fused to the surface of first leaf (Lf1) along the edge in *ad1-224* mutants. (k) SEM showing a macrohair fused to epidermal cells in *ad1-224* mutants. Scale bars in J and K, 100μm. (l) SEM images of immature ears in wild type and *ad1-ref*. The glume of adjacent spikelets are fused in *ad1-ref* mutants. Right image is higher magnification of the area framed in left panel. Scale bars, 500μm. Arrows point to regions of fusion events.

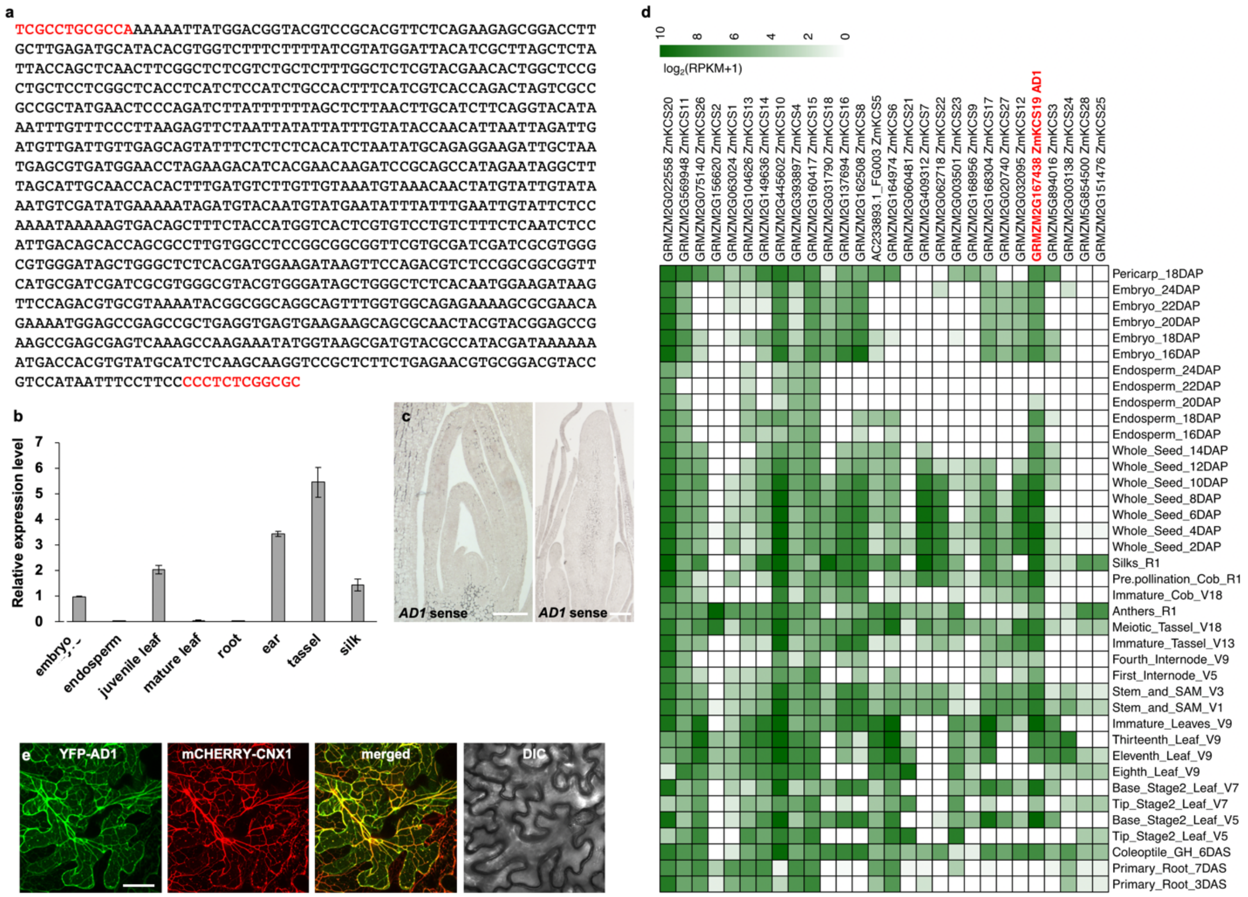

Figure S2. Expression analysis of the *KCS* gene family in maize.

(a) Sequence of the insertion in the *ad1-ref* allele at position +947. In red, partial coding sequence. (b) Quantitative RT-PCR of *AD1* in different maize tissues. The y axis shows the fold change relative to embryo expression levels. Error bars show SD, n=3. (c) *In situ* hybridizations of *AD1* sense control probe in embryo (left panel) and tassel (right panel). Scale bar, 200 μm. (d) RNA-seq expression levels of *KCS* genes in different tissues, from Stelpflug et al. 2016. (e) Confocal images of YFP-AD1 shows co-localization with the ER marker mCHERRY-CNX1 in *N. benthamiana* leaf epidermal cells. Scale bars, 50μm.

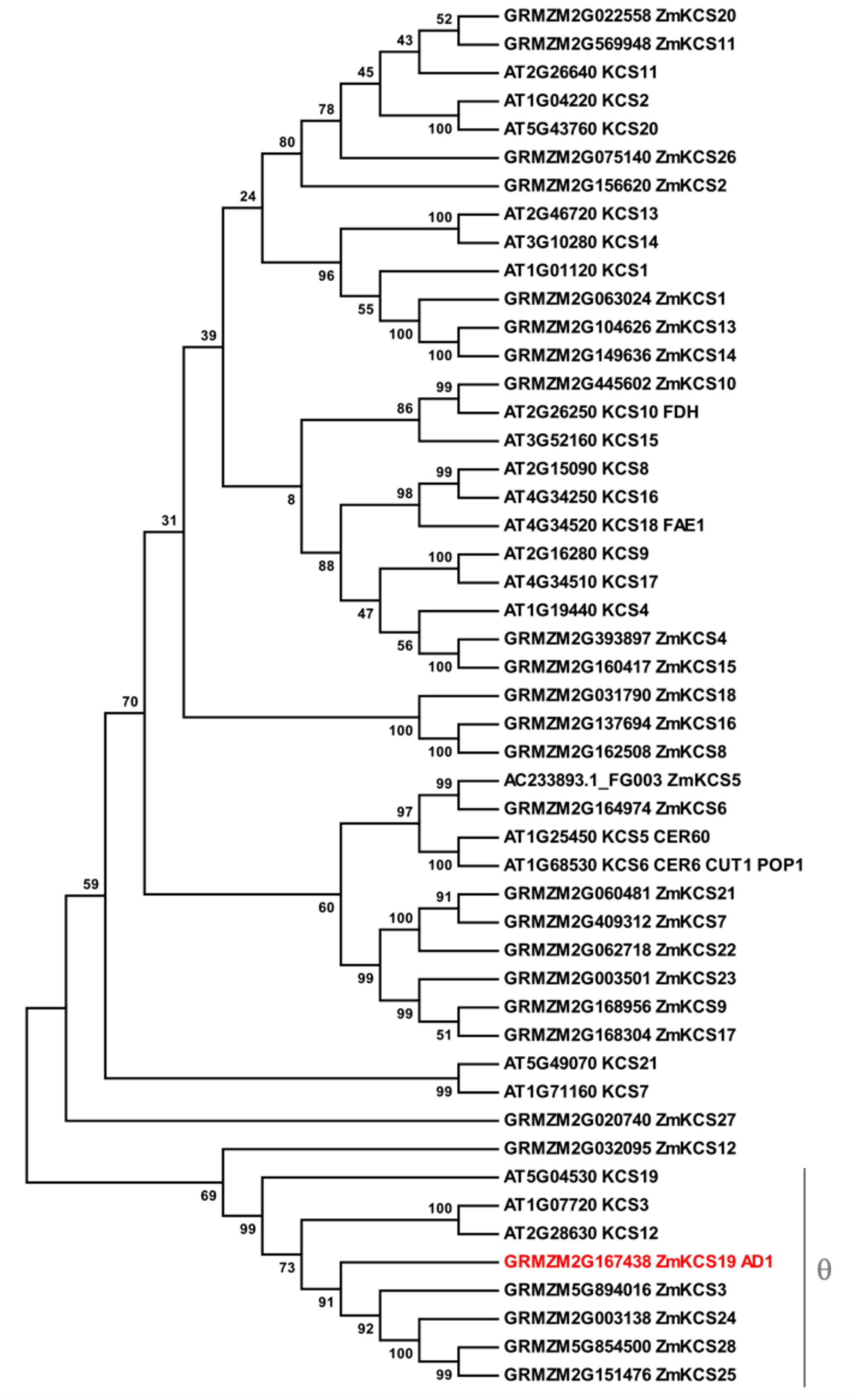

Figure S3. Neighbor-joining phylogenetic tree of maize (GRMZM) and Arabidopsis (AT) KCS family proteins. Numbers represent reliability levels based on 1000 boostrap replications.

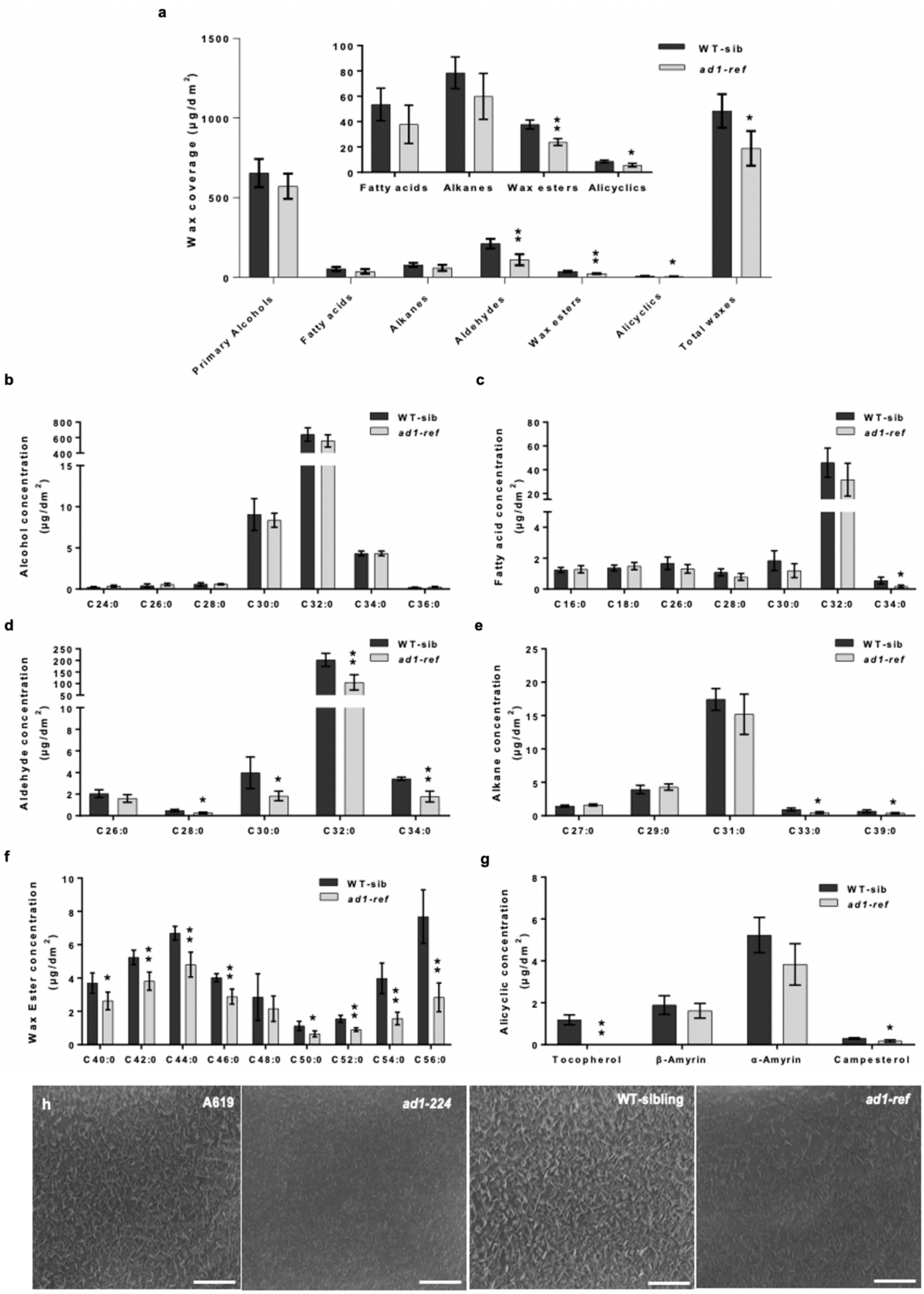

Figure S4. Cuticular wax analysis in *ad1-ref* mutants.

(a) Total wax coverage and amount of each wax class in *ad1-ref* mutants and wild type leaves. The inset shows the less abundant wax classes at a different scale to more clearly visualize significant differences. (b-g) Concentration of individual components in each wax class. primary alcohol (b), fatty acid (c), aldehyde (d), alkane (e), wax ester (f) and alicyclic (g). Means of 4 replicates and SD are reported. *$p<0.05$; **$p<0.01$, Student's test. (h) SEM images of epicuticular wax crystals on the third leaves in A619, *ad1-224*, WT-sibling and *ad1-ref* samples used for the analysis. Scale bars, 5μm.

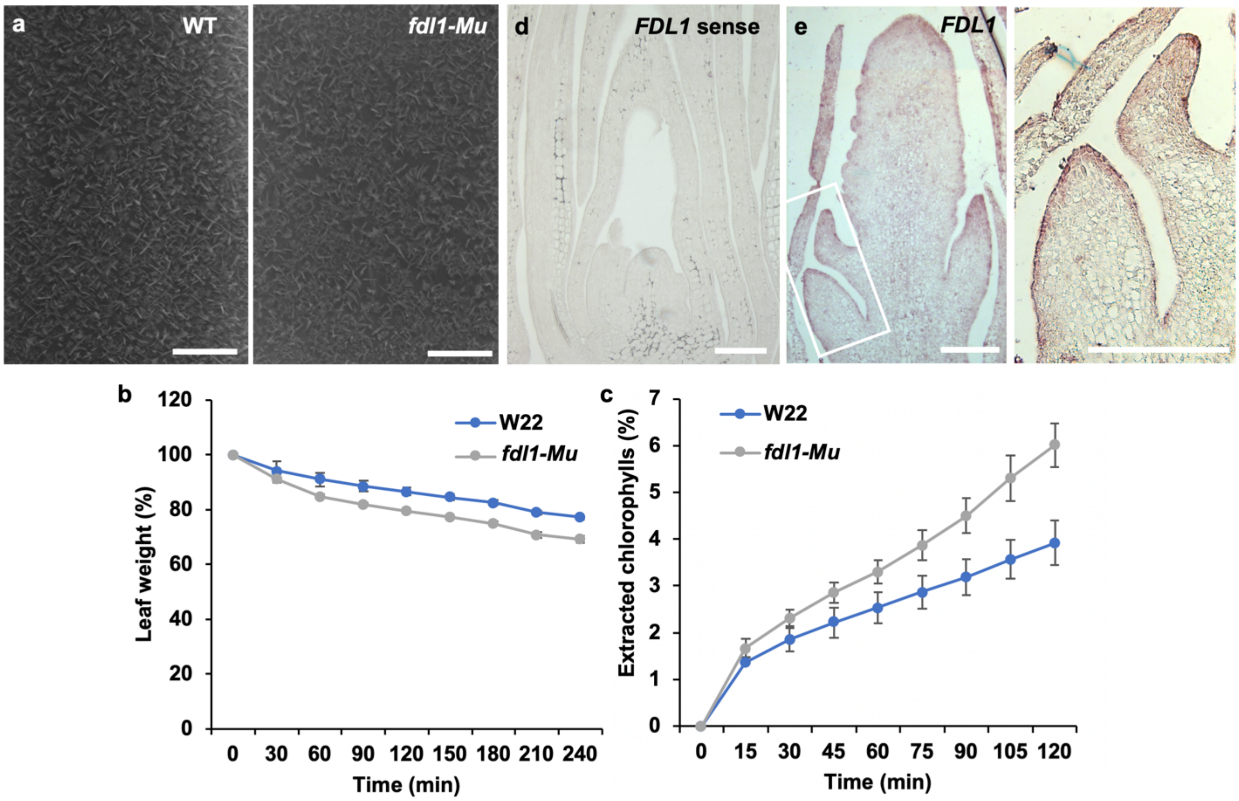

Figure S5. Analysis of *FDL1* function.

(a) SEM images of epicuticular wax crystals on the third leaves in wild type and *fdl1-Mu*. Scale bars, 5μm. (b) Water loss assays. Error bars show SD, n=3. (c) Chlorophyll leaching assays. Error bars show SD, n=3. (d,e) mRNA *in situ* hybridizations using *FDL1* sense control (d) and antisense probes (e). Expression pattern in embryo (d) and tassel (e); the right image is a higher magnification of the area framed in the left panel. Scale bars, 200μm.

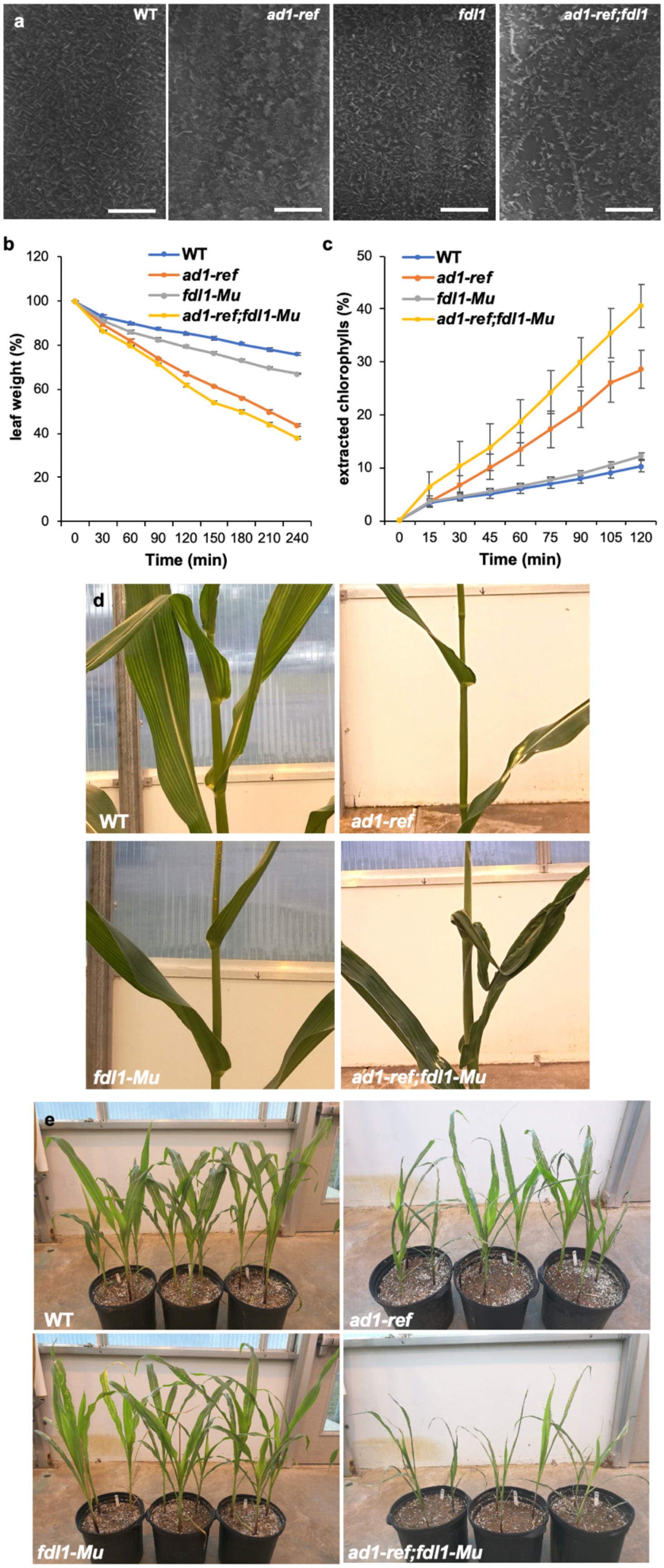
a
WT
ad1-ref
fdl1
ad1-ref;fdl1
b
leaf weight (%)
Time (min)
WT
ad1-ref
fdl1-Mu
ad1-ref;fdl1-Mu
c
extracted chlorophylls (%)
Time (min)
WT
ad1-ref
fdl1-Mu
ad1-ref;fdl1-Mu
d
WT
ad1-ref
fdl1-Mu
ad1-ref;fdl1-Mu
e
WT
ad1-ref
fdl1-Mu
ad1-ref;fdl1-Mu

Figure S6. Double *ad1-ref;fdl1-Mu* mutant analysis.

(a) SEM images of epicuticular wax crystals on the third leaves in wild type, *ad1-ref*, *fdl1-Mu* and *ad1-ref*;*fdl1-Mu*. Scale bars, 5μm. (b) Water loss assays of dark-acclimated plants. Error bars show SD, n=3. (c) Chlorophyll leaching assays. Extracted chlorophyll contents at individual time points were expressed as percentages of that at 24 h after initial immersion. Error bars show SD, n=3. (d) Mature leaf fusion defects in single and double *ad1 fdl1* mutant adult plants. (e) Phenotype of single and double mutant plants exposed to drought stress.

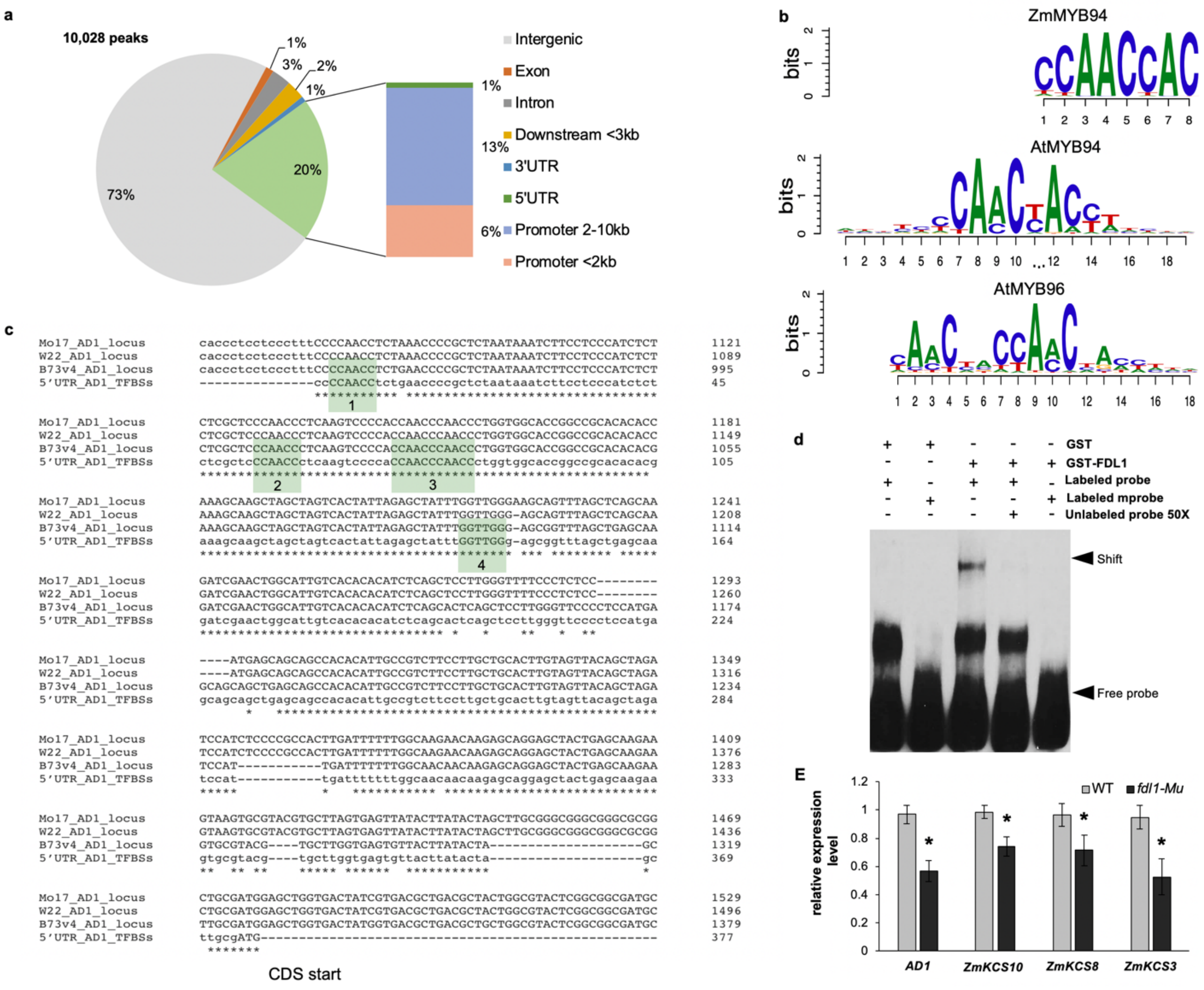

Figure S7. FDL1 DNA binding analysis.

(a) Distribution of FDL1 peaks within gene features. (b) Comparison of DAP-seq identified motifs for ZmMYB94 and AtMYB94 and AtMYB96 (O'Malley et al., 2015). ZmMYB94 and AtMYB94 show highly similar motifs with minor nucleotide variants. (c) Alignment of *AD1* 5'UTR region from maize reference genome inbred line B73 and two additional inbred lines, Mo17 and W22. Region containing CCAACC motifs (highlighted in green; numbered) are conserved across inbreds. (d) EMSA showing GST-FDL1 directly binds to the promoter of *AD1*. Mutation of three of the four CCAACC motifs shown in (c) abolishes the interaction. Competition with unlabeled probe shows specificity of interaction. (e) Comparison of *AD1* and selected KCS genes expression levels between wild type and *fdl1-Mu*. The maize *UBIQUITIN* gene was used as reference. Values are means ± SD (n=3). *p<0.001, Student's two-tail t-test.

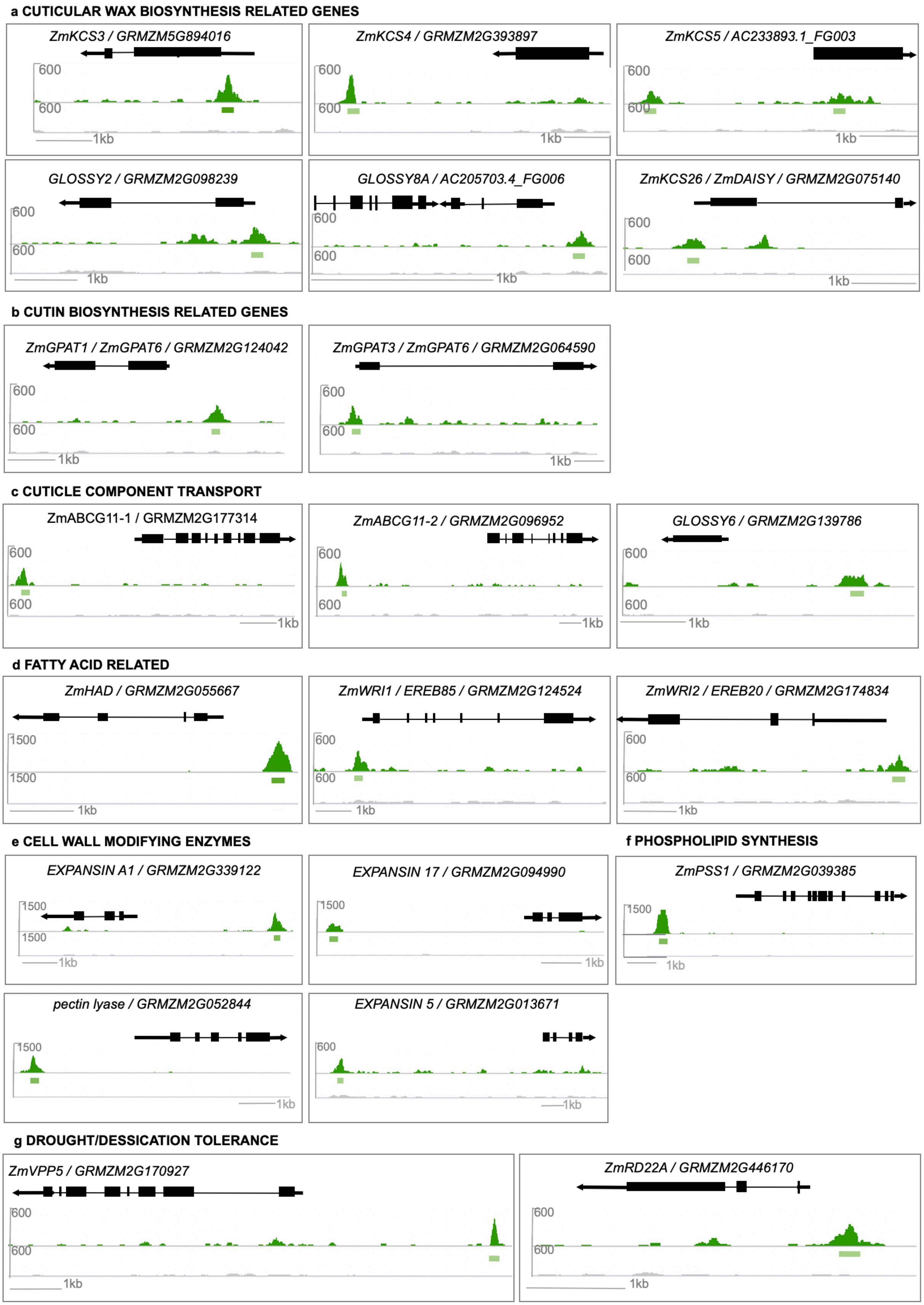
a CUTICULAR WAX BIOSYNTHESIS RELATED GENES
ZmKCS3 / GRMZM5G894016
ZmKCS4 / GRMZM2G393897
ZmKCS5 / AC233893.1_FG003
GLOSSY2 / GRMZM2G098239
GLOSSY8A / AC205703.4_FG006
ZmKCS26 / ZmDAISY / GRMZM2G075140
b CUTIN BIOSYNTHESIS RELATED GENES
ZmGPAT1 / ZmGPAT6 / GRMZM2G124042
ZmGPAT3 / ZmGPAT6 / GRMZM2G064590
c CUTICLE COMPONENT TRANSPORT
ZmABCG11-1 / GRMZM2G177314
ZmABCG11-2 / GRMZM2G096952
GLOSSY6 / GRMZM2G139786
d FATTY ACID RELATED
ZmHAD / GRMZM2G055667
ZmWRI1 / EREB85 / GRMZM2G124524
ZmWRI2 / EREB20 / GRMZM2G174834
e CELL WALL MODIFYING ENZYMES
EXPANSIN A1 / GRMZM2G339122
EXPANSIN 17 / GRMZM2G094990
pectin lyase / GRMZM2G052844
EXPANSIN 5 / GRMZM2G013671
f PHOSPHOLIPID SYNTHESIS
ZmPSS1 / GRMZM2G039385
g DROUGHT/DESSICATION TOLERANCE
ZmVPP5 / GRMZM2G170927
ZmRD22A / GRMZM2G446170

Figure S8. Genome browser screenshots of FDL1 binding.

(a) Genome browser screenshots showing location of FDL1 binding events in several *KCS* and other cuticular wax related biosynthesis genes. Screenshots showing FDL1 binding in cutin biosynthesis related genes (b), cuticular transporters (c), genes related to fatty acid biosynthesis and regulation (d), genes encoding cell wall modifying enzymes (e), a gene regulating phospholipid biosynthesis (f), and genes involved in drought or desiccation tolerance (g). In all panels, 200bp peak regions are shown as green intensity bars under the green FDL1 binding peaks. A negative control sample (affinity tag only) is shown as a gray colored peak track under the green FDL1 peak track.

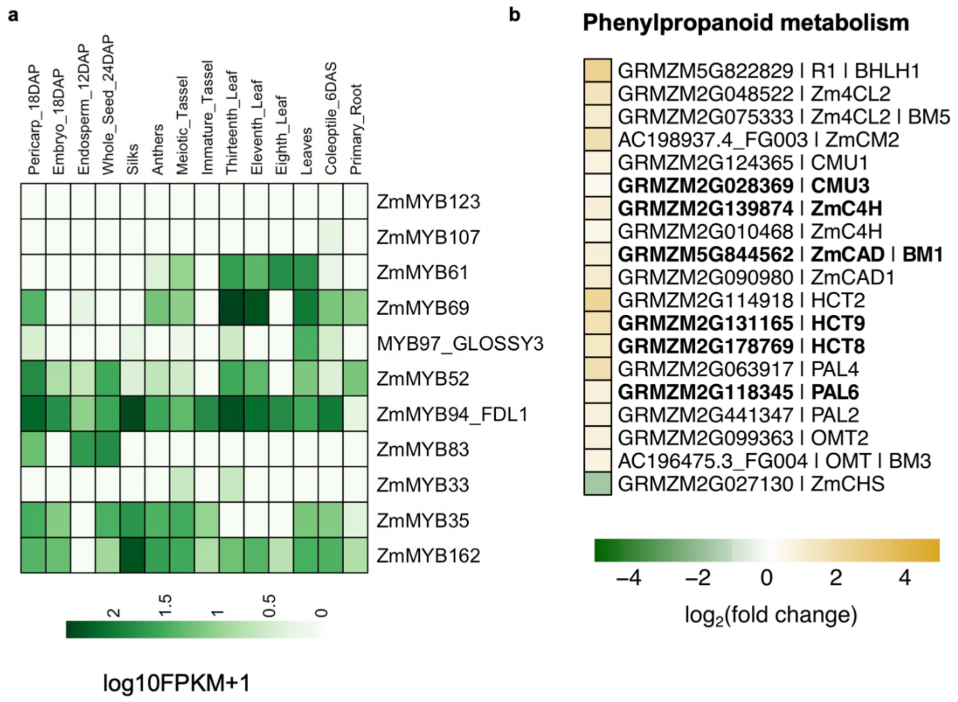

Figure S9. Analysis of *FDL1* expression and binding targets.

(a) *FDL1/ZmMYB94* and closely related *MYB* transcription factors are expressed in many tissues throughout development. *ZmMYB162* is directly bound by FDL1 and up-regulated in *fdl1-Mu* coleoptiles. *ZmMYB83* and *ZmMYB123* are directly bound by FDL1 in DAP-seq. Tissue specific RNA-seq data from Stelpflug et al. (b) Differentially expressed genes in *fdl1-Mu* mutant coleoptiles related to phenylpropanoid metabolism. Genes that were directly bound by FDL1 in DAP-seq are shown in bold.

PRIMERS	SEQUENCE 5'-3'	PURPOSE
ad1-224	For: TGTCAGTCATGGATGCTGGATTACG<br>Rev: CATGAAGGTGCGCTTGTTGCCCGGGTA	dCAPS KpnI - genotyping
ad1-ref	For: TGTCAGTCATGGATGCTGGATTACG<br>Rev: CTCAGCTAGCATTGTTGTGCTCACC	genotyping
fdl1-Mu	For: GAGTACACGAGCAATCCGCAA<br>Rev: CGCCAGGTTGATGTCCGTCT	genotyping
UBI-RT	For: GAGTGCCCCAACGCCGAGTG<br>Rev: CTACGCCTGCTGGTTGTAGACGTA	Real-time PCR
AD1-RT	For: TCTCCAACCCATTCATGGACAA<br>Rev: TTGAGACGTTGAGTTGAGCAGCT	Real-time PCR
ZmKCS10-RT	For: ATGGACGCCTAGCACGTACCT<br>Rev: GGTGAAGTGCAGCACTGCACT	Real-time PCR
ZmKCS8-RT	For: GAGACCACCTACAAGTTCGCTGA<br>Rev: GCTGATGATGAGAAATCAATCATGT	Real-time PCR
ZmKCS3-RT	For: GGAGGACTGCATCGACCAGTA<br>Rev: TCGTTAACCCATCCGAACTTGT	Real-time PCR
AD1-antisense-Probe	For: GAATTCGGCCGTCAAGGCCACCCTAGCTGCTCAACTCAACGT<br>Rev: AGTCGACGGCCCATGAGGCCCAGGACGACACTGACTGATTGACT	*In situ*
FDL1-antisense-Probe	For: GAATTCGGCCGTCAAGGCCAGTGTCCAATCGATCAAGCTCAA<br>Rev: AGTCGACGGCCCATGAGGCCACTTGCACAAGGGGATCTACGTA	*In situ*
AD1-sense-Probe	For:<br>AGTGAATTCGGTAATACGACTCACTATAGGGCTCCCTAGCTGCTCAACTCAAC<br>Rev: AGTCGACGGCCCATGAGGCCCAGGACGACACTGACTGATTGACT	*In situ*
FDL1-sense-Probe	For:<br>AGTGAATTCGGTAATACGACTCACTATAGGGCTGTGTCCAATCGATCAAGCTC<br>Rev: AGTCGACGGCCCATGAGGCCACTTGCACAAGGGGATCTACGTA	*In situ*
AD1-EMSA-Probe	For: GACCCATGTCCATGATGTGGTAT<br>Rev: CCAAATAGCTCTAATAGTGACTA	EMSAs
AD1-Pro-mut1	For: TTCAAGTCCCCATTTTTTTTTTTTTGGTGGCACCGGCCGCACAC<br>Rev: AAAAAAAATGGGGACTTGAAAAAAAAAAAGCGAGAGAGATGGGAGG	EMSAs
AD1-Pro-mut2	For: CCCTTTTTTTTTTTTCTGAACCCCGCTCTAATAAATCTTCCT<br>Rev: GGTTCAGAAAAAAAAAAAAGGGAGGAGGGTGGAGGGTGGTT	EMSAs
pGreenII-*AD1*Pro	For: TCGAGGTCGACGGTATCGATCCACACAATCGAGCACGACAT<br>Rev: CGGCCGCTCTAGAACTAGTGCGCAAGCTAGTATAAGTAACA	Transient Transcription Assay
pRI101-*MYB94*	For: AAGTTCTTCACTGTTGATACATGGTGAGGCCGCCGTGCTG<br>Rev: AGTTGTTGATTCAGAATTCGCTAGAAGGGGTAATCCATGG	Transient Transcription Assay

Table S1. List of primers used in this study.

ZmName	geneID	peak location	peak distance from TSS	Peak Signal Value	log2FC in fdl1-Mu coleoptiles	Arabidopsis homolog	description
**Cuticular wax and fatty acid elongation**							
ADHERENT1, KCS19	GRMZM2G167438	Promoter (<=1kb)	0	33	#N/A	*KCS12,KCS19*	3-ketoacyl-CoA synthase
ZmKCS3	GRMZM5G894016	5' UTR	643	47	#N/A	*KCS12,KCS3*	3-ketoacyl-CoA synthase
ZmKCS4	GRMZM2G393897	Downstream (2-3kb)	5130	44	#N/A	*KCS4*	3-ketoacyl-CoA synthase
ZmKCS5	AC233893.1_FG003	Promoter (2-3kb)	-2617	28	#N/A	*CER6,CUT1,G2,KCS6,POP1*	3-ketoacyl-CoA synthase
		Exon	330	33.2			
ZmKCS26	GRMZM2G075140	Promoter (<=1kb)	0	30	-0.554471	*KCS2*	3-ketoacyl-CoA synthase
GLOSSY8A	AC205703.4_FG006	Promoter (<=1kb)	-627	40	#N/A	*ATKCR1,KCR1,YBR159*	beta-ketoacyl reductase
GLOSSY26	GRMZM2G481843	Promoter (<=1kb)	0	35.8	-0.396167	*CER10,ECR,TSC13*	3-oxo-5-alpha-steroid 4-dehydrogenase family protein
GLOSSY2	GRMZM2G098239	Promoter (<=1kb)	0	34	#N/A	*CER2,VC-2,VC2*	HXXXD-type acyl-transferase family protein
ZmCER3	GRMZM2G029912	Intron	214	35	-0.524724	*CER3,FLP1,WAX2,YRE*	Fatty acid hydroxylase superfamily
GLOSSY6	GRMZM2G139786	Promoter (1-2kb)	-1851	39	-0.518466	*AT1G09310*	Protein of unknown function, DUF538
**Cutin**							
ZmBDG	GRMZM2G030636	5' UTR	122	44	-1.12516	*BDG1,BODYGUARD*	alpha/beta-Hydrolases superfamily protein
		Promoter (<=1kb)	0	97			Zm
ZmGPAT3	GRMZM2G064590	Promoter (<=1kb)	0	37	#N/A	*ATGPAT5,ATGPAT6*	glycerol-3-phosphate acyltransferase
ZmGPAT1	GRMZM2G124042	Promoter (<=1kb)	-805	35	#N/A	*GPAT6*	glycerol-3-phosphate acyltransferase 6
**Wax and cutin transport**							
ABCG11-2	GRMZM2G177314	Promoter (2-3kb)	-2639	31	-0.432011	*ABCG11,COF1,DSO,WBC11*	white-brown complex homolog protein 11
ABCG11-5	GRMZM2G096952	Promoter (6-7kb)	-6563	50	#N/A	*ABCG11,COF1,DSO,WBC11*	white-brown complex homolog protein 11
**Fatty Acid synthesis**							
ZmHAD	GRMZM2G055667	Promoter (<=1kb)	-773	176	-0.354572	*HAD*	Thioesterase superfamily protein
**TFs related to fatty acid/cuticle formation**							
EREB85,WRI1	GRMZM2G124524	Promoter (<=1kb)	0	36	0.626041	*AIL5,CHO1,EMK,ASML1,ATWRI1*	AINTEGUMENTA-like 5, EREB TF
EREB20,WRI2	GRMZM2G174834	Promoter (<=1kb)	-115	31	#N/A	*AIL5,CHO1,EMK,ASML1,ATWRI1*	AINTEGUMENTA-like 5, EREB TF
EREB143	GRMZM2G141219	Promoter (<=1kb)	-110	24.6	#N/A	*ASML1,ATWRI1,ADAP*	ARIA-interacting double AP2 domain protein, EREB TF
ZmNFXL2	GRMZM2G139369	Promoter (1-2kb)	-1881	68	#N/A	*ATNFXL2*	sequence-specific DNA binding transcription factor
**Phospholipid Synthesis**							
ZmPSS1	GRMZM2G039385	Promoter (2-3kb)	-2572	141.2	#N/A	*AT1G71150*	phosphatidyl serine synthase family protein
**Vesicle trafficking and secretory pathway**							
SEC14	GRMZM2G704053*	Promoter (3-4kb)	-3358	31	0.435357	*AT1G75170*	Sec14p-like phosphatidylinositol transfer family protein
ZmSYP121	GRMZM2G414540	Promoter (<=1kb)	-20	74	NA	*PEN1,SYP121, AT3G11820*	syntaxin of plants 121
**Signaling**							
ZmGSO1	GRMZM2G313643	Promoter (<=1kb)	0	32	0.540328	*GSO1,SGN3, AT4G20140*	LRR receptor-like serine/threonine-protein kinase GSO1
ZmGSO1	GRMZM2G172429	NA	NA	NA	-0.92111	*GSO1,SGN3, AT4G20140*	LRR receptor-like serine/threonine-protein kinase GSO1

Table S2. List of genes involved in cuticle formation directly bound by FDL1 in DAP-seq.

REFERENCES

O'Malley RC, Huang SSC, Song L, Lewsey MG, Bartlett A, Nery JR, Galli M, Gallavotti A, Ecker JR. 2016. Cistrome and Epicistrome Features Shape the Regulatory DNA Landscape. *Cell* **165**(5): 1280-1292.

Stelpflug SC, Sekhon RS, Vaillancourt B, Hirsch CN, Buell CR, de Leon N, Kaeppler SM. 2016. An Expanded Maize Gene Expression Atlas based on RNA Sequencing and its Use to Explore Root Development. *Plant Genome* **9**(1); doi.org/10.3835/plantgenome2015.04.0025.
